# Herbivore functions in the hot-seat: Resilience of *Acanthurus triostegus* to marine heatwaves

**DOI:** 10.1371/journal.pone.0318410

**Published:** 2025-01-31

**Authors:** Taylor Souza, Jeroen Brijs, Leon Tran, Larry Crowder, Jacob L. Johansen

**Affiliations:** 1 Hopkins Marine Station, Stanford Oceans, Stanford Doerr School of Sustainability, Pacific Grove, CA, United States of America; 2 Hawaiʻi Institute of Marine Biology, University of Hawaiʻi at Mānoa, Kāne’ohe, HI, United States of America; MARE – Marine and Environmental Sciences Centre, PORTUGAL

## Abstract

Herbivorous fishes play a crucial role in the conservation of coral reefs threatened by thermal stress (*e*.*g*., marine heatwaves and long-term ocean warming) by helping to maintain reefs in a coral-dominated state via the removal of algae. However, as thermally sensitive ectotherms, rising thermal stress may also pose a serious threat to these fishes and the critical ecosystem functions they deliver. Here we evaluate the consequences of thermal stress on the capacity of a common herbivorous coral reef fish (*Acanthurus triostegus*) to control finely filamentous matrices of *Caulerpa sertularioides* and *C*. *verticillata* algae in Hawai*ʻ*i, by characterizing *in-vivo* changes in metabolic demands, diurnal foraging rates, activity patterns and individual condition in a laboratory setting during winter (24.0±0.1°C), summer (27.5±0.1°C), and at the peak of a representative marine heatwave, (31.0±0.1°C). Rising temperatures caused significant increases in standard metabolic rate (from ~135 O_2_ kg^-1^ h^-1^ in winter to 224 O_2_ kg^-1^ h^-1^ at the peak of a marine heatwave), but not in the proportion of time spent active (~83–96%) or foraging (~2.4 bites min^-1^). Consequently, *A*. *triostegus* gained body mass during summer and winter, but lost ~0.8% body mass per day during the marine heatwave. Given marine heatwaves can last for weeks to months, these results indicate that while herbivorous coral reef fishes may continue to remove algae during periods of thermal stress, their ability to control many macroalga may be limited due to precipitous reductions in individual performance. Therefore, in addition to algal types, the thermal sensitivity in herbivorous reef fishes will need to be considered for the successful implementation of coral-algal management strategies in a warmer world.

## Introduction

Coral reefs, often referred to as the “rainforest of the sea,” occupy less than 1% of the ocean floor yet are home to over 25% of all marine life, making them among the most biodiverse and productive ecosystems on Earth [1]. Given their ecological significance, it is crucial to understand the impact of forceful agents of disturbance such as marine heatwaves (MHWs) [2]. MHWs, defined as “discrete prolonged anomalously warm water events with sea-surface temperatures exceeding the 90^th^ percentile threshold calculated across 30 years and persisting for at least 5 days” [3], are known to have increasingly devastating and long-lasting impacts on ecosystem resilience and services [4–7]. The extreme warming associated with MHWs has caused widespread coral devastation globally [8–10] including recurring mass coral bleaching events [10–13], with notable impacts in the Pacific [8, 14, 15]. Alarmingly, these disturbances have been increasing in frequency, intensity, severity, and duration over the past century and are projected to continue to do so throughout the next century [5, 6, 16]. It is anticipated that the escalation of MHWs may eventually lead to the irreversible loss of many coral reef ecosystems worldwide [5, 8, 16, 17].

In light of these projections, understanding the ecological impacts of MHWs on coral reef ecosystems becomes of utmost importance. The severe or prolonged disturbances caused by MHWs can lead to a collapse of habitat structure and a transition from coral to algal dominance [18, 19]. Since the shift from coral to algal dominance fundamentally alters the ecosystem, the role of herbivorous reef fishes is regarded to be essential for the conservations of coral reefs in a warmer future [19–21]. By removing algae that may otherwise hinder the settlement, growth, and survival of corals and coral recruits [18, 19, 21–23], herbivorous reef fishes help prevent and mitigate transitions from coral to algal-dominated states [18, 24, 25]. Therefore, reductions in their abundance may directly undermine reef resilience. In fact, herbivorous coral reef fishes, including ’browsers’ (*i*.*e*., species that consume fleshy, rapidly growing macroalgae encroaching on established coral), ’grazers’ (*i*.*e*., species that feed on algal turf covering coral settlement surfaces), and ’scrapers’ (*i*.*e*., species that actively scrape the reef matrix to reveal fresh coral settlement areas), are often thought to serve as the final line of defense against uncontrolled algal proliferation [18]. However, most reef fishes are also highly sensitive to rising temperatures [26–28], leading to uncertainty about species health and resilience, including the retention and delivery of herbivorous reef fish functions, as these ecosystems are increasingly exposed to temperatures beyond those for which they have evolved [26, 29, 30].

As ectotherms, the metabolic energy demands and condition of herbivorous reef fishes is regulated by ambient ocean temperatures [31–34]. Specifically, the minimum oxygen uptake required by an ectotherm to maintain homeostasis (denoted as standard metabolic rate, SMR) [35], typically increases at a rate of 2x for every 10°C rise (defined as a Q_10_) [36, 37]. In thermally sensitive species, including tropical coral reef fishes, SMR often increases at a Q_10_ rate of 2-3x [38], suggesting ocean warming of 3–4°C—such as might occur during a marine heatwave—could increase basal energetic demands for survival by up to 55% [38, 39]. Current projections indicate a global average temperature increase of approximately 2.7°C above preindustrial levels (or about 1.7°C above present day) [16], underscoring the importance of understanding the potential impacts of both chronic warming and acute thermal stress events on these species. Studies have already demonstrated that temperatures just 2–3°C above annual summer maxima can have detrimental effects on multiple traits of tropical coral reef fishes, including swimming, growth, activity, reproduction and foraging patterns [28, 31, 40–44]. Accordingly, rising temperatures pose a potential threat to the retention and function of herbivorous reef fishes as increased energetic demands will necessitate increased energy acquisition (*e*.*g*., by increasing algal foraging rates), reduced energy expenditure (*e*.*g*., by reducing activity) [40, 45, 46] or shift in diets to more energetic items [47]. However, herbivorous reef fishes already spend over 80% of their time feeding during peak summer conditions [48, 49] and are not expected to have the ability to process food more quickly through their digestive system [44, 48, 50–53] suggesting substantial increases in foraging rates may be infeasible.

Given these constraints, there is great uncertainty of how herbivorous reef fishes may cope with rising energetic demand and the resultant impacts on herbivore functions [54, 55]. Therefore, as we look to these organisms to help safeguard reef ecosystems into the future [24, 44, 56], this study aims to investigate the consequences of rising ocean temperatures on the retention and delivery of herbivorous reef fish functions. Using an ecologically important herbivorous reef fish, the convict tang (*Acanthurus triostegus*) and a turf matrix of filamentous green algae (*Caulerpa sertularioides* and *C*. *verticillata*) as model representatives, we hypothesize that as ocean temperatures increase (*i*.*e*., from winter, to summer, to the peak of a MHW), herbivores will show two responses to cope with elevated energy requirements: 1) algal foraging rates will increase, and 2) energy expenditure will reduce (*i*.*e*., minimizing activity time). By investigating these thermal responses, we aim to gain insight into whether an important herbivorous reef fish can maintain its body condition while continuing to remove algae in a warmer future.

## Materials and methods

### Ethical approval

Animal care and all of the experimental procedures described below complied with the ethical standards of the Institutional Animal Care and Use Committee at the University of Hawaiʻi at Mānoa, approved under the permit number 3200. All experiments involving *Acanthurus triostegus* were conducted with the utmost care to minimize distress to the animals. For all trials, no animals were harmed and no animals were euthanized. Following the completion of the experiments, all animals were returned to their capture locations.

### Study species and housing

Based on its broad Indo-Pacific distribution, high abundance and dispersal ability on shallow water reefs throughout the equatorial Pacific [57], *Acanthurus triostegus*, locally known as manini (family Acanthuridae) was selected as the representative herbivorous coral reef fish in this study, which typically feed on fine-filamentous algae [58, 59]. Solitary fish ranging between 25 g– 105 g (representing juvenile and sub-adult populations) were collected by snorkelers using hand nets during the winter (January-March) and summer (July-September) of 2021 and 2022 when sea surface temperatures were 24.0±0.5°C or 27.5±0.5°C, respectively. Fish size was estimated in water using standard underwater techniques, including visual estimation with reference objects or rulers for scale. Upon collection, fish were blotted dry and weighed with an electronic scale (to 0.1g precision), and individuals under 25 g were returned to the water. All fish were collected from reefs surrounding the Hawai*ʻ*i Institute of Marine Biology (HIMB) on Moku o Lo*ʻ*e in Kāne*ʻ*ohe Bay (21.4323°N, 157.788°W) on the eastern coast of O*ʻ*ahu, Hawai*ʻ*i, and transported to the Johansen Fish Resilience Laboratory at HIMB. Individuals were held in groups of 4–6 in large conical holding tanks (height: 86.0 cm; diameter: 82.5 cm; volume: 260 L) containing sections of PVC pipe for shelter. Tanks were continuously supplied with flow-through, filtered, aerated and UV-sterilized seawater and subjected to a 12hr:12hr light:dark photoperiod. Water temperatures were maintained at 24.0±0.1°C during winter and 27.5±0.1°C during summer reflecting mean seasonal sea surface temperatures around Moku o Lo*ʻ*e [60]. This was achieved by either heating tanks with aquarium heaters (TH-08005, Finnex Inc., USA) or cooling tanks using submersible pumps (D08V045CD, Kedsum, China) that were connected to stainless steel coils submerged in a 95 L reservoir held at 5°C by a water chiller (ECO-1 ½ HP Ecoplus, USA). The aquarium heaters and submersible pumps were in turn connected to temperature control relays (WH1436, Willhi, China) that activated when the water temperatures were not within the desired temperature range.

Fish were allowed to acclimate to laboratory conditions for two weeks prior to experimentation and were supplied *ad libitum* with a daily surplus of fresh algal-turf matrix collected around Moku o Lo*ʻ*e (hereafter referred to as green algae) [61]. The matrix was externally identified to be comprised primarily of *Caulerpa sertularioides (<10%) and C*. *verticillate (~90%)* algae, a finely filamentous alga 2–6 cm in length. Thin matrices of these algae were abundant HIMB (pers. obs.), although there is no clear evidence on their status as native or invasive [62, 63], or general abondance throughout Hawai’i. However, it has been reported that species in the genus *Caulerpa* show a high degree of morphological plasticity, allowing these species to adapt to different environments and thus increasing their invasive potential [64, 65]. Indeed, these algae are turf-forming macroalgae, known to spread laterally by producing extensive rhizomes [66] and form low and finely filamentous algal turfs in early growth stages [67]. This family of algae is also known to spread favorably within existing algal turfs helping to anchor fragments and its rhizoids [68–77]. When collected for this experiment, both species of *Caulerpa* were intertwined, creating a turf matrix of fine filamentous algae. The collected algal matrix was kept together for feeding experiments to preserve its structural integrity and provide fishes with food items natural to their environment. *Caulerpa spp*. *are* foraged upon by a wide range of herbivores, but like many invasive algae contain sesquiterpenoid compounds, caulerpin and caulerpicin, with aldehyde or enol acetate functional groups [78–81]. These functional groups are considered non-toxic but show herbivore feeding deterrent properties such as reduced palatability [78].

### Experimental treatments

Fish collected between January and March were assigned to the winter treatment (*i*.*e*., 24.0±0.1°C, n = 22), whereas fish collected between July and September were randomly assigned to either the summer treatment (*i*.*e*., 27.5±0.1°C, n = 22) or the MHW treatment (*i*.*e*., July-September, 31.0±0.1°C, n = 17). All fish were collected within a narrow body mass range, with initial estimates made in the field and precise measurements obtained using an electronic scale in the laboratory (to 0.1g precision), to control for potential body mass effects. The average body mass (± S.E.M) for each season was as follows: winter: 55.6±4.1 g, summer: 52.0±5.4g, and MHW: 46.8±4.5 g (Table 1). ANOVA results confirmed no significant difference in body mass between the groups: winter:summer (*T*_*49*_ = -0.542, *P* = 0.590), winter:heatwave (*T*_*49*_ = -1.427, *P* = 0.480), summer:heatwave: (*T*_*49*_ = -0.736, *P* = 0.590). A MHW was simulated by increasing water temperature for 4 days by ~0.9°C per day from mean summer sea surface temperature (27.5±0.1°C) to the peak of the MHW (31.0±0.1°C), whereafter water temperatures were maintained at the peak of the MHW for three more days before trials began (total experimentation time: winter– 9 days, summer– 9 days, MHW– 12 days) (See S4.3 in S2 Fig) [82]. These conditions closely matched the mean duration (days), maximum heating rate (°C day^-1^), and maximum intensity (°C) for MHWs detected on the reefs within Kāne*ʻ*ohe Bay between 1994–2020, ensuring that MHW simulations equated to the historical MHW conditions of the collection area [60, 82].

**Table 1:** Average body mass (± S.E.M) of fish collected across seasonal treatments: Winter, summer, and marine heatwave conditions.

Winter	Summer	Heatwave
55.6 ± 4.1	52.0 ± 5.4	46.8 ± 4.5

### Evaluating foraging rates and activity

Following acclimation to laboratory conditions, fish were fasted for ~24 h, captured, weighed and measured. To avoid fluctuations in body mass measurements due to wet weight, each fish was gently blotted dry with a soft, absorbent material prior to being weighed on an electronic balance. Fish were handled quickly and carefully to minimize stress and potential harm during this process. Fish were then individually assigned to randomized treatment tanks (identical to the holding tanks and housing conditions described above, with 1 fish per tank), where water temperatures were maintained at either 24.0±0.1°C for the winter treatment group or 27.5±0.1°C for the summer and MHW treatment groups. In an effort to standardize feeding and maintain water quality, fish were supplied daily with a surplus supply of ~5–10 g of green algae (i.e. a finely filamentous matrix of *Caulerpa spp*.) attached to a PVC pipe with a rubber band at 08:30 HST, which was subsequently removed along with any floating or demersal food remnants at 16:30 HST. Fish were allowed three days to resume ‘normal’ foraging behavior before trials began, as pilot trials revealed that foraging rates and activity levels of *A*. *triostegus* typically reached a stable plateau within that period of time (see S1 Fig). Fish were then video-recorded throughout the day (*i*.*e*., between 09:30–09:40, 11:10–11:20, 12:50–13:00, 14:30–14:40, and 16:10–16:20 HST) using a 4K surveillance camera system at 15 frames per second (N842 Series, Lorex Technology Inc., Canada) (see detailed figure in S4.1 in S2 Fig). Fish in the winter and summer treatment groups were video-recorded for three days at their respective temperatures, while fish in the simulated MHW treatment group were subjected to a simulated MHW and then video-recorded for the final three days when water temperatures were maintained at the peak of a MHW (see *Experimental treatments* for details). At the end of each trial, fish were fasted for ~24 hours in preparation for intermittent-flow respirometry (see *Evaluating standard metabolic rate* for details) to avoid the confounding effects of specific dynamic action on whole-animal aerobic metabolic rates [83]. Once the fish had been removed from the treatment tanks to be weighed, measured, and placed into respirometers, the entire system was scrubbed and rinsed with clean seawater.

Video-recordings were visually analyzed for foraging rates (*i*.*e*., defined as the number of bites per minute an individual took from the green algae) and activity levels (*i*.*e*., defined as the proportion of time that the fish were active in the treatment tank (*i*.*e*., swimming around the tank and/or feeding on the algae) [40, 84]. Mean foraging rates and activity levels were then calculated from the three successive days of video-recordings for each treatment., while daily changes in body mass were determined by dividing the percent gain or loss of initial body mass (*i*.*e*., [final body mass–initial body mass] / initial body mass × 100) by the number of days in the trial.

### Evaluating standard metabolic rate

Oxygen uptake rates (*Ṁ*O_2_) were obtained using intermittent-flow respirometry and used to determine SMR (standard metabolic rate) (*i*.*e*., the metabolic rate of a fasted and resting individual) as an estimate of changes in basal energy demand [85–87]. Individuals of *A*. *triostegus*, a social species, were transferred from their treatment tanks into one of four cylindrical acrylic respirometers (volume of 0.594 L, 14 cm long and 7 cm Ø or volume of 1.600 L, 22 cm long and 10 cm Ø depending on size of individual). The respirometers were submerged in an experimental tank (length: 97 cm, width: 53 cm, height: 37 cm) supplied with flow-through, filtered, fully aerated and UV-sterilized seawater under a 12 hr:12 hr light:dark photoperiod. To accommodate their social nature, each fish was placed in a respirometer that allowed visual access to other fish in the tank. Water temperatures were maintained within 0.1°C of winter, summer, and peak simulated MHW treatment temperatures. Water was continuously mixed within each respirometer using an in-line submersible pump (AD20P-1230E, DollaTek, USA) within a recirculation loop to ensure a homogenous concentration of oxygen throughout the respirometer [87] (see detailed figure in S4.2 in S2 Fig). Automated flush pumps intermittently refreshed the water within each respirometer according to the flush cycles set for determining SMR (‘Flush’ = 5 min, ‘Wait’ = 1 min, and ‘Measure’ = 1 min) in the AquaResp software (v3.04, www.aquaresp.com). This ensured that oxygen levels in the respirometers always remained above 80% air saturation [87]. The partial pressure of oxygen in the water within each respirometer was measured continuously at 1 Hz using a fiber optic oxygen sensor mounted in the recirculation loop where the flow is sufficient to ensure a rapid response time of the sensor [87]. The optic sensor from each respirometer was connected to a 4-channel Firesting Optical Oxygen Meter (Pyro Science, Germany), which in turn were connected to a PC that logged the data. Mass-specific *Ṁ*O_2_ values were automatically calculated by the AquaResp software from the linear decline in the partial pressure of oxygen during each measurement period (*i*.*e*., when the flush pumps were off). Only *Ṁ*O_2_ calculations with an R^2^ ≥ 0.95 were kept for further analysis. Fish remained in the respirometer for at least 24 hours during which *Ṁ*O_2_ values were continuously determined. Pilot trials on this species and previous studies on similar reef fish species revealed that this time period is more than sufficient for the individual to reach a stable resting state [85, 88]. Following the completion of the trial, fish were released back into the wild at the approximate location of capture. To account for background respiration, we used linear regression over time, subtracting bacterial ṀO2 measurements from empty respirometers taken before and after each respirometry trial from all ṀO2 data, with background respiration contributing 3.9% ± 2.9 (winter), 3.2% ± 2.6 (summer), and 5.7% ± 2.0 (heatwave) (mean ± standard deviation). To minimize background respiration rates, all equipment was disinfected with a 1% bleach solution, thoroughly rinsed with freshwater, and allowed to dry before starting subsequent trials. The 20% quantile method was used to calculate SMR, as this method yielded the most consistent estimates based on visual inspection of *Ṁ*O_2_ and the cumulative variance of the mean lowest normal distribution [85].

### Data analysis

All statistical analyses were performed using R (R Development Core Team, v4.2.1 2022) and detailed descriptions of the statistical procedures are provided in the supplementary information (see S1 File – raw script; S1 Table – raw data). Body mass was compared between treatment groups (i.e., winter, summer, and marine heatwave) using paired t-tests. Two modeling approaches were used and compared here. The first approach used separate linear regressions for each response variable (i.e., standard metabolic rate (SMR), foraging rate, percent body mass change, activity). The models for SMR, foraging rate, and activity were fit with treatment *(i*.*e*., winter, summer, and marine heatwave) and final body mass, and the interaction between treatment and body mass. For percent body mass change, only treatment was included as a predictor variable. To adhere to the assumptions of normality and homoscedasticity, forage rate, SMR, and activity data were transformed using a square root transformation, and log10 transformation for negative skew, and Box-Cox transformation, respectively.

The second modeling approach implemented a linear mixed effects model (LMM) for SMR, foraging rate, and percent body mass change and a generalized linear model (GLM) with a gamma distribution and identity link function for the activity data. For both the LMM and GLM, the predictor terms included treatment (*i*.*e*., winter, summer, and marine heatwave), final body mass, and the interaction between treatment and final body mass, and individual fish ID was included as a random intercept for the LMM. Due to the unequal scales of SMR, percent body mass change, and foraging rate data, a routine z-score standardization was applied to accurately estimate standard errors and model coefficients. Non-normal SMR and foraging rate data were transformed using a Yeo-Johnson transformation to satisfy the assumptions of normality and homoscedasticity. The resulting models from these two approaches were compared using corrected Akaike Information Criterion (AICc) to select the final models to be used for further analysis, where the models with the lowest AICc values were deemed the best fit models to the data. Overall, the first modeling approach yielded the lowest AICc values and were selected for the final analysis (See S1 File).

Model fit was assessed using adjusted R-squared values for all models. The assumption of normality was assessed using Shapiro-Wilks test of normality and visual inspection of quantile-quantile plots. Homoscedasticity was assessed using the parametric Bartlett’s and the non-parametric Levene’s tests for equality of variances along with visual inspection of model residuals plotted against model fitted values. Hypothesis testing was conducted using planned multiple comparison t-tests, comparing mean SMR, bite rate (bites min^-1^), activity (duration of active time outside the shelter) and percent body mass gain or loss per day across the three treatment groups (winter, summer, and MHW). False discovery rate corrections were used to control for family-wise error rate [89]. These metrics aimed to detect statistical differences in SMR, bite rate (bites min^-1^), activity (duration of active time outside the shelter), and the percentage of body mass gain or loss per day. The comparisons were conducted among the three treatments (winter, summer, and simulated MHW), under the expectation that as SMR increased, bite rate (bites min^-1^) would increase or energy expenditure would decrease. Q_10_ coefficients (*i*.*e*., the rate of increase in metabolic oxygen demand across temperatures) [90] were calculated to compare the effect of temperature on SMR between seasons (Eq 1) where R_i_ represents metabolic rate *i* and T_i_ represents temperature *i* [36]. All data is presented as mean + S.E.M. unless otherwise stated.


$$Q_{10}={\frac{R_{2}}{R_{1}}}^{\frac{10}{T_{2}-T_{1}}}$$ (Eq 1)

## Results

Between treatment groups, there was no significant difference in body mass (winter:summer: t_49_ = -0.736, P > 0.05; winter:MHW: t_49_ = -1.427, P > 0.05; summer:MHW: t_49_ = -0.736, P > 0.05). From the regression analysis, the interaction between treatment and body mass was significant only for activity (foraging rate: F_2_ = 1.273, P > 0.05; SMR: F_2_ = 2.273, P > 0.05; activity: F_2_ = 4.729, P < 0.05). Activity generally increased with mass in the summer groups and decreased with mass in the winter and MHW groups, but these overall trends were statistically insignificant. The interaction term between body mass and treatment was removed from the foraging rate, SMR, and activity models. There was no effect of body mass on SMR (F1,37 = 4.0204, P > 0.05) or foraging rate (F₁,₄₈ = 0.311, P > 0.05).

**Fig 1:**
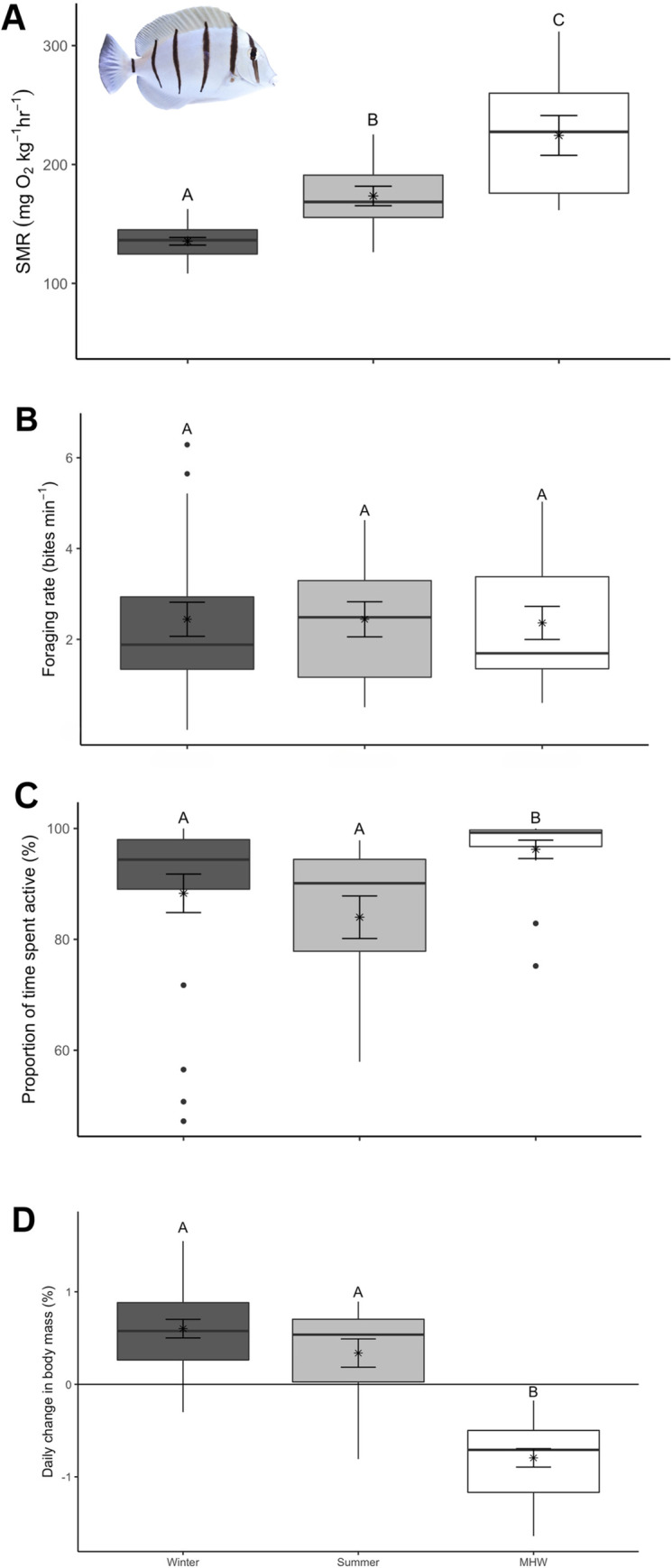
Acanthurus triostegus response to seasonal temperature treatments. (A) Standard metabolic rate (SMR), (B) foraging rates, (C) activity levels, and (D) daily percent body mass change of *A*. *triostegus* during winter (24.0±0.1°C), summer (27.5±0.1°C), and at the peak of a simulated MHW (31.0±0.1°C). Raw data were used to construct the box and whisker plots that display the mean ± S.E.M. (‘x’± error bars), median (horizontal line within box), 25th and 75th percentile (lower and upper horizontal lines of box), minimum and maximum values (whiskers), and the outliers (black circles, values that exceed a distance of 1.5 times the interquartile range below or above the 25th and 75th percentile, respectively). Significant differences (*p*<0.05) between treatments are represented by different upper-case letters.

*Acanthurus triostegus* exhibited a clear temperature-dependent increase in standard metabolic rate (F₂,₃₇ = 34.155, P < 0.001; winter: 135 ± 3 O_2_·kg^-1^·h^-1^; summer: 173 ± 8 O2·kg-1·h-1; MHW: 224 ± 17 O2·kg-1·h-1; Fig 1A), with SMR notably higher during summer and MHW conditions compared to winter (winter:summer: *T*_*37*_ = 4.820, *P* < 0.0001; winter:MHW: *T*_*37*_ = 8.060, *P* < 0.0001; summer:MHW: T_37_ = 3.083, P = <0.05). Overall, SMR approximately doubled for every 10°C increase in water temperature, equating to Q_10_ values = 2.03 (winter:summer), 2.06 (winter:heatwave), 2.09 (summer:heatwave).

Contrary to expectations, no change in foraging rates were observed across temperatures (winter:summer–*T*_*48*_ = 0.8.6, *P* >0.05; winter:MHW–*T*_*48*_ = -0.054, *P* >0.05; summer:MHW–*T*_*48*_ = -0.223, *P*>0.05), as foraging rates remained stable at ~2.4 bites min^-1^ (Fig 1B).

*A*. *triostegus* demonstrated a marked increase in activity levels during the MHW compared to winter (*T*_*48*_ = -2.696, *P* < 0.05) and summer (*T*_*48*_ = -3.965, *P* < 0.001) treatments but not between winter and summer (*T*_*48*_ = 1.654, *P* > 0.05). Mean activity values ranged between ~88 ± 3.47% in winter, ~83 ± 3.83% in summer, and ~96 ± 1.65% during the MHW (Fig 1C).

Significant differences in the percent body mass change of *A*. *triostegus* were observed between winter:MHW (*T*_*49*_ = -9.139, *P* < 0.05) and summer:MHW (*T*_*49*_ = -6.495, *P*<0.05), but not between winter:summer (*T*_*49*_ = -1.595, *P*>0.05). Mean percent body mass increased during winter and summer at 0.6 ± 0.1% day^-1^ and 0.3 ± 0.2% day^-1^, respectively, while it significantly decreased during the MHW at -0.8 ± 0.1% day^-1^ (Fig 1D).

## Discussion

This study investigated the impacts of rising temperature on rates of energetic demand (*i*.*e*., SMR), acquisition (*i*.*e*., foraging rates) and expenditure (*i*.*e*., activity levels) of an ecologically important herbivorous coral reef fish foraging on a finely filamentous matrix of *Caulerpa sertularioides* and *C*. *verticillata*. While herbivorous coral reef fishes are expected to increase foraging rates or reduce activity to meet rising metabolic demands brought on by ocean warming, this premise has to our knowledge not been explicitly tested. Contrary to our expectations, we demonstrate that despite rising metabolic demands, *A*. *triostegus* does not increase foraging rates, and instead, increases levels of activity as temperatures increase. Consequently, the temperature driven increase in metabolic demands, alongside steady feeding rates and increased activity levels, resulted in a significant reduction in body mass when exposed to a simulated MHW. Since feeding rates remained constant, algae consumption rates would not decrease directly due to temperature. However, the thermally induced reductions in the condition of *A*. *triostegus* combined with expectations of faster growth rates of many algae in warmer waters [91] could indirectly affect their long-term ability to control algae. This could have important implications for our reliance on herbivorous functions in a warming world, with potential downstream consequences for reef resilience and the trophic structure [55].

Energetic demands of *A*. *triostegus*, here evaluated as oxygen consumption, increased by ~28% from winter to summer (Q_10_ of 2.0) and an additional ~29% from summer to the peak of a simulated MHW (Q_10_ of 2.1). These thermally induced increases in energetic demand were similar to that observed in a range of other coral reef fish species (*e*.*g*., Q_10_ of 1.1–5.7 (29–33°C) [31, 92, 93] and matched the global mean projections for most marine organisms of Q_10_ = 2–3 [39, 86, 94]. To overcome these thermally driven energetic demands many species are expected to increase energy acquisition. However, contrary to the anticipated increase, the foraging rates of *A*. *triostegus* remained consistent across temperatures. Previous studies of herbivore responses to temperature have shown comparable results. Some herbivorous fish, especially parrotfishes, exhibit increased foraging rates with changing latitudinal or seasonal temperatures [44, 50, 51, 53]. However, at temperatures outside their optimal range, these rates can plateau or even decrease [50, 95]. For instance, feeding rates of *Acanthurus bahianus* have been shown to decrease more rapidly than metabolic rates when temperatures drop, suggesting an inability to maintain feeding efficiency at suboptimal temperatures. This diminished feeding rate in cooler waters highlight a temperature-dependent physiological constraint [96]. Here, the examined MHW conditions are beyond the temperatures under which most reef fishes have evolved and a plateau in feeding rates suggests temperature-dependent physiological constraints may be mirrored at temperatures above optimum.

Emerging studies suggest that the diet of herbivores may influence their thermal tolerance due to changes in e.g. fatty acid composition needed for cardiac function [97]. Accordingly, the nutritional quality of *C*. *sertularioides* and *C*. *verticillata*, and their specific metabolic requirements for digestions, might not only affect the energy availability but also the physiological capacity of *A*. *triostegus* to withstand elevated temperatures. In this context, it is important to consider that fish experiencing higher energetic demands during MHWs may be compounded by the concurrent inability to increase their foraging rates to boost intake. The resulting nutritional and energetic deficiencies, combined with higher SMR, could easily limit energy allocation to processes like growth and reproduction as seen in this study. Indeed, the fact that fish in summer conditions maintained body weight, while MHW fish could not, suggest that it was no longer possible to balanced increased metabolic demands with digestive and assimilative efficiencies. Future research should explore these potential trade-offs, as digestive constraints may further complicate the relationship between foraging, temperature, and energy balance.

The reason for unaltered foraging rates in herbivorous fishes is uncertain but may be caused by foraging mode and/or the algal type examined. Unlike piscivores, which may have the flexibility to opt for larger and more frequent meals, herbivores adopt a fermentation-based digestion process resembling an assembly line [52, 58, 98]. Many species are thought to operate at near maximum foraging capacity under current day temperatures, typically spending >80% of their time in this activity [48], potentially making further increases in foraging rates infeasible [52]. Interestingly, herbivorous fish in Hawai*ʻ*i consist of multiple families, each with different digestive strategies. Conducting similar studies on other functional groups of herbivorous reef fish could reveal varying responses to thermal stress due to differences in their digestive physiology and their roles in coral-algal interactions. For instance, unicornfish (*Naso spp*.) and parrotfish (*Scarus spp*.) are abundant herbivores in Hawai*ʻ*i that primarily rely on foregut fermentation. In contrast, surgeonfish (*Acanthurus spp*.) and chubs (*Kyphosus spp*.) exhibit hindgut fermentation [99]. The foregut fermentation process, involving microbial breakdown of plant material early in digestion, may allow these herbivores to better extract nutrients from algae [99]. This could theoretically leave some herbivores less impacted by ocean warming. Additionally, Specific Dynamic Action (SDA), which represents the energy used for meal digestion and assimilation likely escalates under increased temperatures, further complicating the energy budget during MHWs [83]. As SDA varies with diet composition, understanding its impact under different thermal conditions could provide insights into how *A*. *triostegus* and similar species manage their energy needs when foraging cannot be increased. This point underscores the need for comparative studies across different herbivore guilds to better understand their ecological roles and resilience to environmental changes.

In addition to foraging rates, the algae matrix examined here, *Caulerpa sertularioides* and *C*. *verticillata* contain terpenoid metabolites thought to diminish attractiveness to herbivores [78, 100]. It is possible, that these chemicals may diminish palatability or impede rapid digestion in herbivores [78, 100], potentially inhibiting increases in herbivore foraging rates even in scenarios where increased foraging is necessary for condition and survival. Indeed, this scenario seems to be supported by our data, as the recorded foraging rates of ~2.4 bites min^-1^ are substantially below typical foraging rates observed for *A*. *triostegus* on other algae species in the wild (typical ~10–50 bites min^-1^ [101–103]. Here, *A*. *triostegus* maintained body mass by foraging on these algae during winter and summer alike, but experienced significant body mass loss during a MHW. As a result, our findings indicate that less attractive algae species may be the first to escape top-down herbivore grazing due to ocean warming, raising concerns about steadily diminished control of problematic algae as oceans warm.

It is important to acknowledge that the experiment was conducted in an aquarium setting, which inherently isolates the fish from natural competitors and predators. This controlled environment could potentially result in altered activity rates compared to those observed in natural settings. Additionally, the preference for certain algae may change as temperatures increase, as different diet items can influence the thermal tolerance due to changes in e.g. fatty acid composition needed for cardiac function [97]. Furthermore, as food was only available between 08:30 and 16:30 HST, this schedule may not fully encapsulate the natural feeding periods of some coral reef fish, which may feed more actively from sunrise to sunset [104]. In nature, individuals will also have to spend time searching for food and recurrently hide from predator, none of which was issues in our predator-free system with *ad-libitum* food. However, individuals might still have faced an added challenge of feeding during a shorter time period than possible in the wild, with potentially lower total food intake than theoretically possible. Regardless, the reduced conditions of fishes during the MHW compared to winter and summer conditions reveal that MHWs can drastically change the status quo for feeding patterns of herbivorous fishes, likely demanding either faster or longer feeding periods each day to maintain condition. While the broader implications for different algal species remain uncertain, our data suggest less attractive algae, such as *C*. *verticillata*, could face unique challenges as the effectiveness of top-down algal control may diminish as oceans warm. Dietary flexibility in response to changing thermal conditions may also play a key role in the ability of these fishes to maintain fitness and condition during such events.

The activity data for *A*. *triostegus* revealed that individuals did not attempt to conserve energy during marine heatwaves (MHWs), the only condition where they experienced a substantial change in temperature from their accustomed ambient conditions. Instead, the fish spent 83 to 96% of their time outside the shelter, actively swimming and foraging, with significant increases in activity observed during MHW conditions. Given their constant need to feed, reducing activity levels may not be a practical strategy. The increased activity during elevated MHW temperatures may suggest avoidance behavior, where *A*. *triostegus* attempted to escape the warmer conditions. This situation places *A*. *triostegus* and other herbivores in a challenging position, where they must constantly forage without any opportunity to conserve energy, while their current foraging strategy cannot match the thermally induced elevations in energy demand.

Given the inability to increase food intake or reduce activity, a viable third strategy for herbivorous coral reef fishes to cope with periods of ocean warming may involve relocating to more thermally suitable habitats. Specifically, poleward migration or movement to cooler deeper regions of the reef located below the thermocline could allow a species to remain within optimal thermal conditions [34, 105]. However, while scientific studies have demonstrated that some coral reef fish species can successfully migrate to more thermally optimal habitats during extreme temperatures [34, 105–108], most adult coral reef fishes do not possess the ability to partake in poleward migration as this phenomenon predominantly occur during the dispersal of larval juvenile fishes. Similarly, refuge seeking in deeper sections of the reef may be equally implausible, as MHWs commonly span the entire water column of coral reef environments (including those in our study sites in Hawai*ʻ*i [13].

The mismatch between energy demands and energy acquisition caused a precipitous loss of body mass with potential downstream consequences for condition. A 0.8% decrease in body mass on a daily basis is undoubtedly unsustainable, and our findings therefore suggest that these fishes may only be able to continue their trait of top-down algal control for a limited period of time during severe MHWs. If this pattern holds true in the wild, a decrease in the condition and local abundance of herbivorous reef fishes during prolonged warming periods might lead to reduced rates of algal removal, particularly those with herbivore defenses. This reduction could in turn increase the likelihood of ecosystems transitioning from coral dominance to algal dominance [19, 20]. This is particularly worrying, as MHWs are projected to increase significantly in frequency, intensity, duration, and expansiveness in the years to come [5, 6, 16].

Overall, our findings indicate that herbivorous coral reef fishes may be more adversely affected by rising ocean temperatures than previously expected, as elevated energetic demands coupled with limitations in energy acquisition severely compromised the condition of a common herbivorous reef fish and/or its ability to help control algal proliferation, at least for some species of algae. Considering the association between rising ocean temperatures and the increased likelihood of transitions from coral dominance to algal dominance, foraging adaptations that underpin the ability of herbivores to mitigate these risks may be in jeopardy. Our results reveal an important relationship between energy demands, foraging patterns, and the condition of herbivorous reef fish in the context of global change ecology. They also highlight that effective herbivore conservation and management strategies, aimed at supporting the resilience of tropical marine ecosystems, would benefit from further research in this field. In particular, more study is needed to determine whether the patterns observed in the present study are consistent across different algal types and the various functional traits of other herbivorous fishes.

## Supporting information

S1 Fig Foraging rate and activity levels of *Acanthurus triostegus* during pilot trials. **A)** Foraging rates and **(B)** activity levels of *A*. *triostegus* during pilot trials to evaluate the period of time required for both these parameters to stabilize, which typically occurred after 3 days. Data are presented as mean±95%C.I. and pilot trials were conducted at 24.0±0.1°C.
(PDF)

S2 Fig Experimental Set-Up: S4.1. **Experimental Set-Up.** Detailed diagram of holding tanks for the study on Acanthurus triostegus. The setup includes 4K surveillance cameras operating at 15 frames per second, N842 Series from Lorex Technology Inc., Canada, and two 4–6" PVC structures provided for shelter. An aquarium heater, TH-08005 from Finnex Inc., USA, and a cooling tank with stainless steel coils connected to a water chiller, ECO-1 ½ HP from Ecoplus, USA, are used for temperature regulation. Temperature control is maintained using WH1436 relays from Willhi, China. **S4.2. Respirometer Set-Up.** Detailed respirometer set-up for A. triostegus to evaluate Standard Metabolic Rate. **S4.3. Experimental Timeline.** An expanded timeline for the experimental treatments evaluating the resilience of Acanthurus triostegus to marine heatwaves. The timeline includes details of acclimation periods, ramping of tank temperatures for the MHW treatment group, and daily protocols for feeding, video recording, and conducting intermittent-flow respirometry. Fish were subjected to incremental temperature increases up to 31.1 ±0.1°C and provided ~5–10 g of algae daily to assess the impact of thermal stress on their metabolic demands and foraging behavior.
(PDF)

S1 Table. Raw data Raw data used in the statistical analyses within ’Herbivore functions in the hot-seat: Resilience of Acanthurus triostegus to marine heatwaves’. (CSV)

S1 File. R-script R-script for data analysis within Herbivore functions in the hot-seat: resilience of *Acanthurus triostegus* to marine heatwaves. (PDF)
