# Supplementary material for: Herbivore functions in the hot-seat: Resilience of Acanthurus triostegus to marine heatwaves
Source: PLoS One. 2025 Jan 31;20(1):e0318410. doi: 10.1371/journal.pone.0318410 (PMC11785343; doi:10.1371/journal.pone.0318410)
Supplement: S1 Fig — A) Foraging rates and (B) activity levels of A. triostegus during pilot trials to evaluate the period of time required for both these parameters to stabilize, which typically occurred after 3 days. Data are presented as mean±95%C.I. and pilot trials were conducted at 24.0±0.1°C. (PDF) [file pone.0318410.s001.pdf]

Supplementary Information 1
Article title: Herbivore functions in the hot-seat: Resilience of *Acanthurus triostegus* to marine heatwaves
Journal name: Coral reefs
Author names: Taylor Souza, Jeroen Brijs, Leon Tran, Larry Crowder, and Jacob L. Johansen
Corresponding author: Taylor Souza, Stanford Doerr School of Sustainability, taysouza@stanford.edu

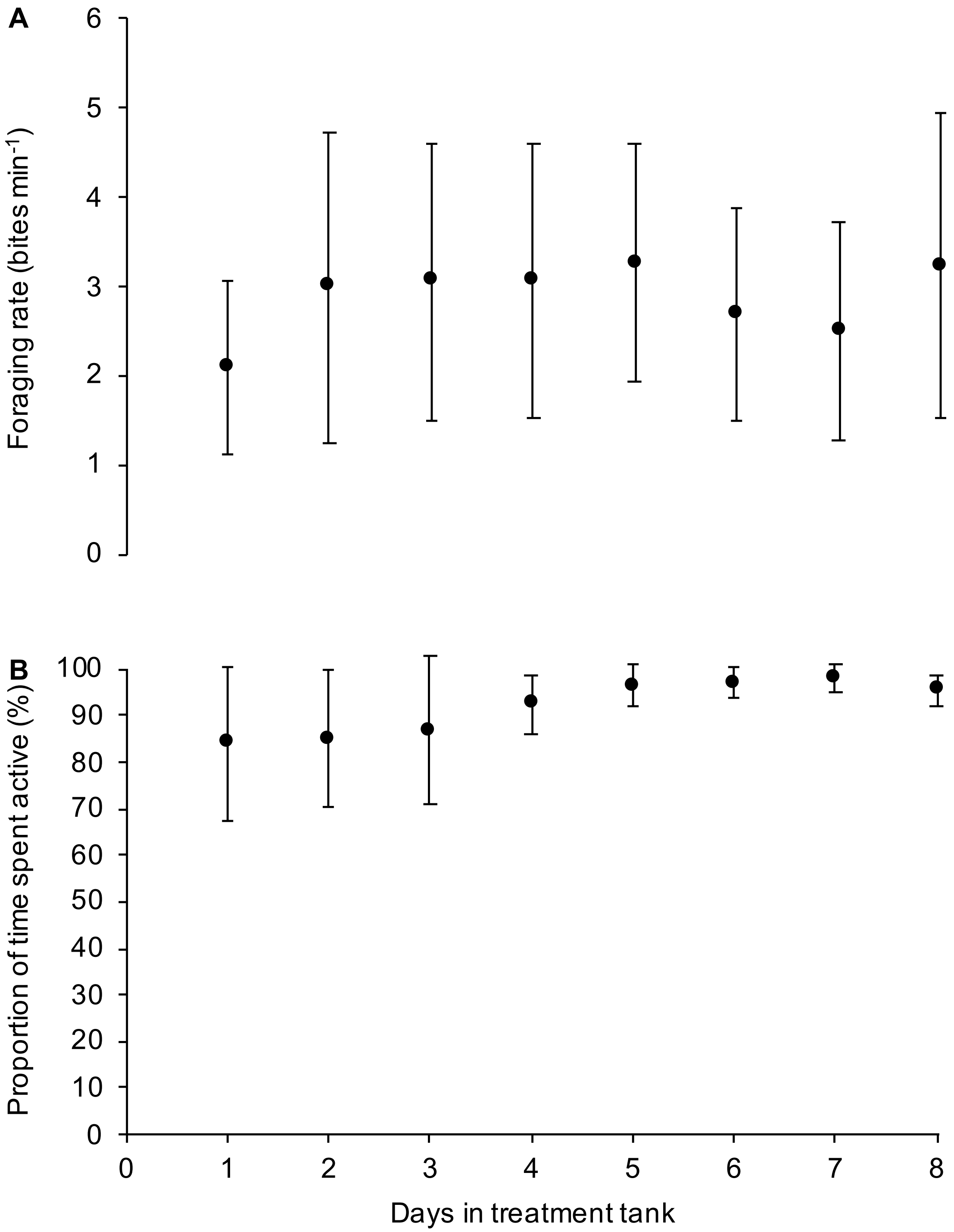

Supp. Info. 1 (A) Foraging rates and **(B)** activity levels of *A. triostegus* during pilot trials to evaluate the period of time required for both these parameters to stabilize, which typically occurred after 3 days. Data are presented as mean±95%C.I. and pilot trials were conducted at 24.0±0.1°C
