# Supplementary material for: Herbivore functions in the hot-seat: Resilience of Acanthurus triostegus to marine heatwaves
Source: PLoS One. 2025 Jan 31;20(1):e0318410. doi: 10.1371/journal.pone.0318410 (PMC11785343; doi:10.1371/journal.pone.0318410)

S4.1 Experimental Set-Up

Detailed diagram of holding tanks

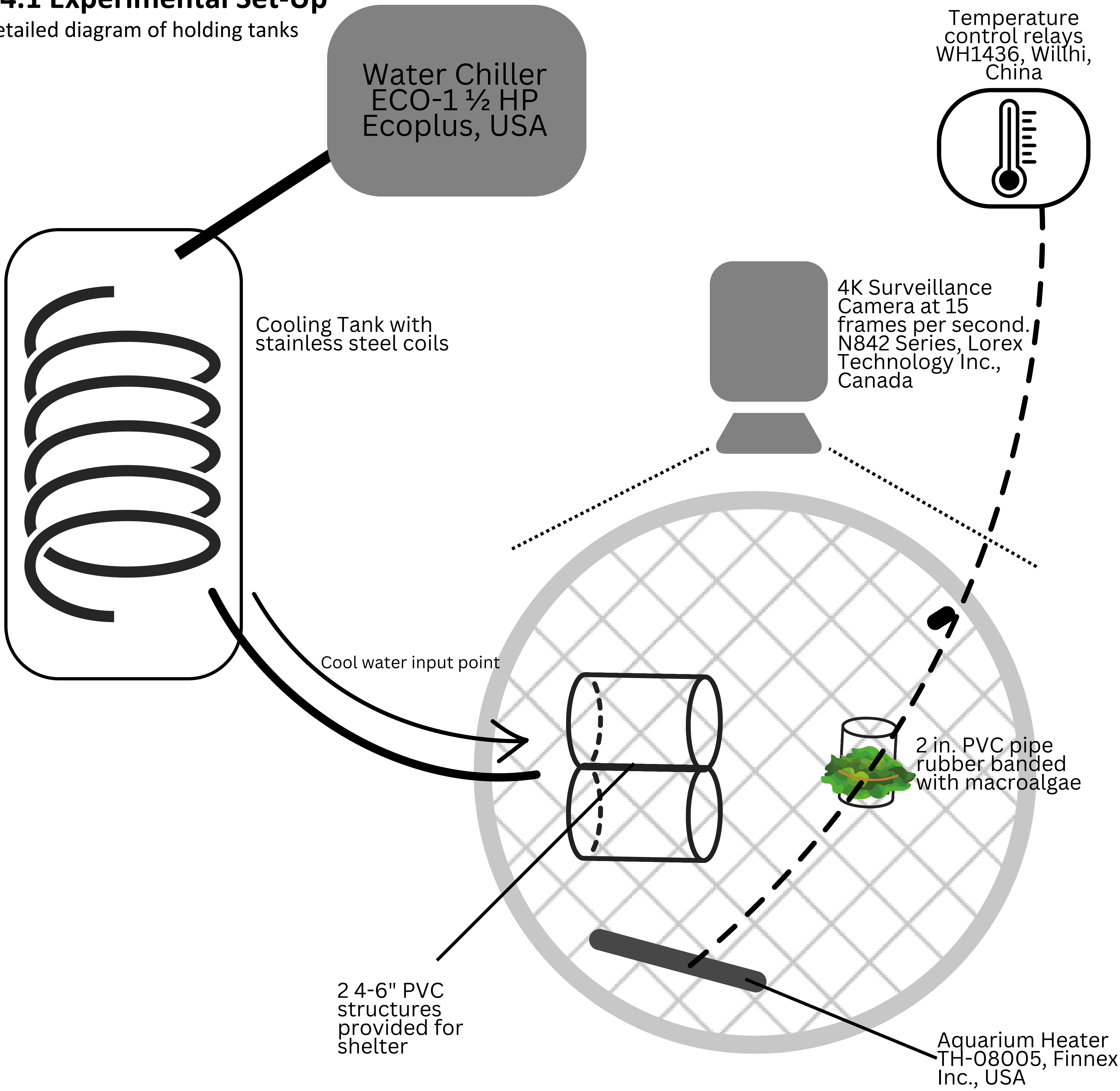

S4.2 Respirometer Set-Up

Detailed respirometer set-up for *A. triostegus* to evaluate Standard Metabolic Rate.

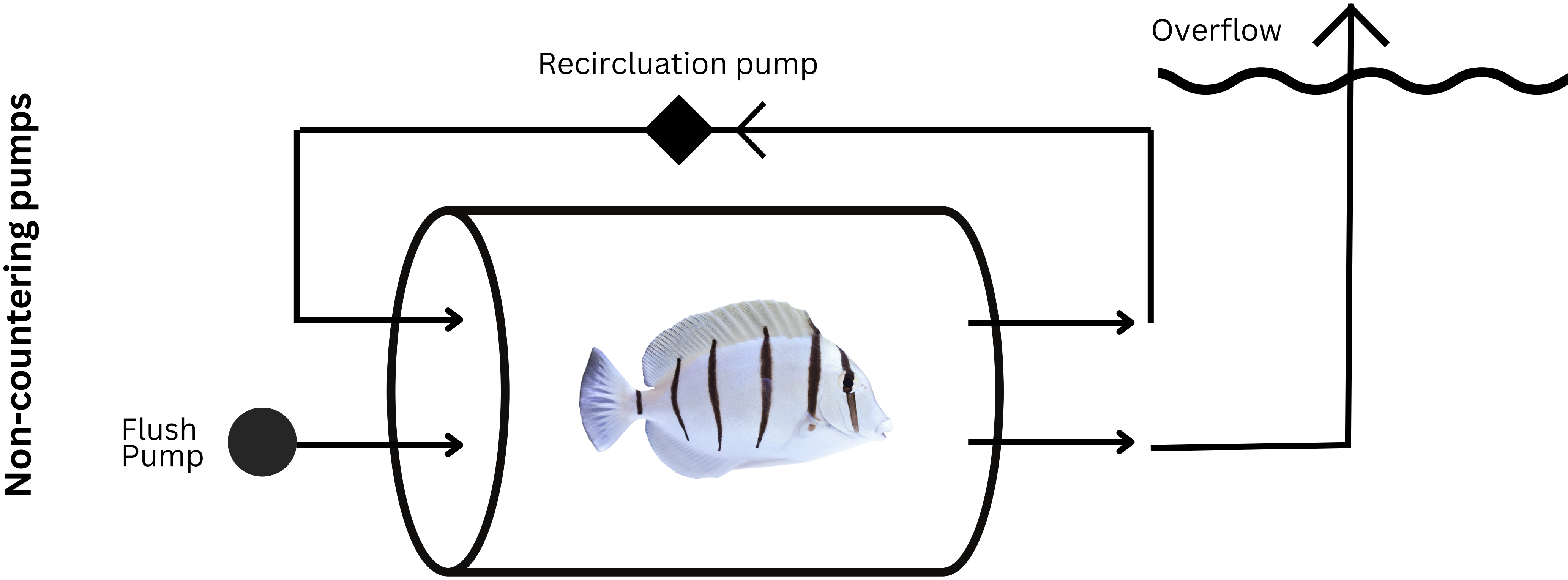

S4.3 Experimental Timeline

Experimental Timeline for Herbivore functions in the hot-seat: resilience of Acanthurus triostegus to marine heatwaves

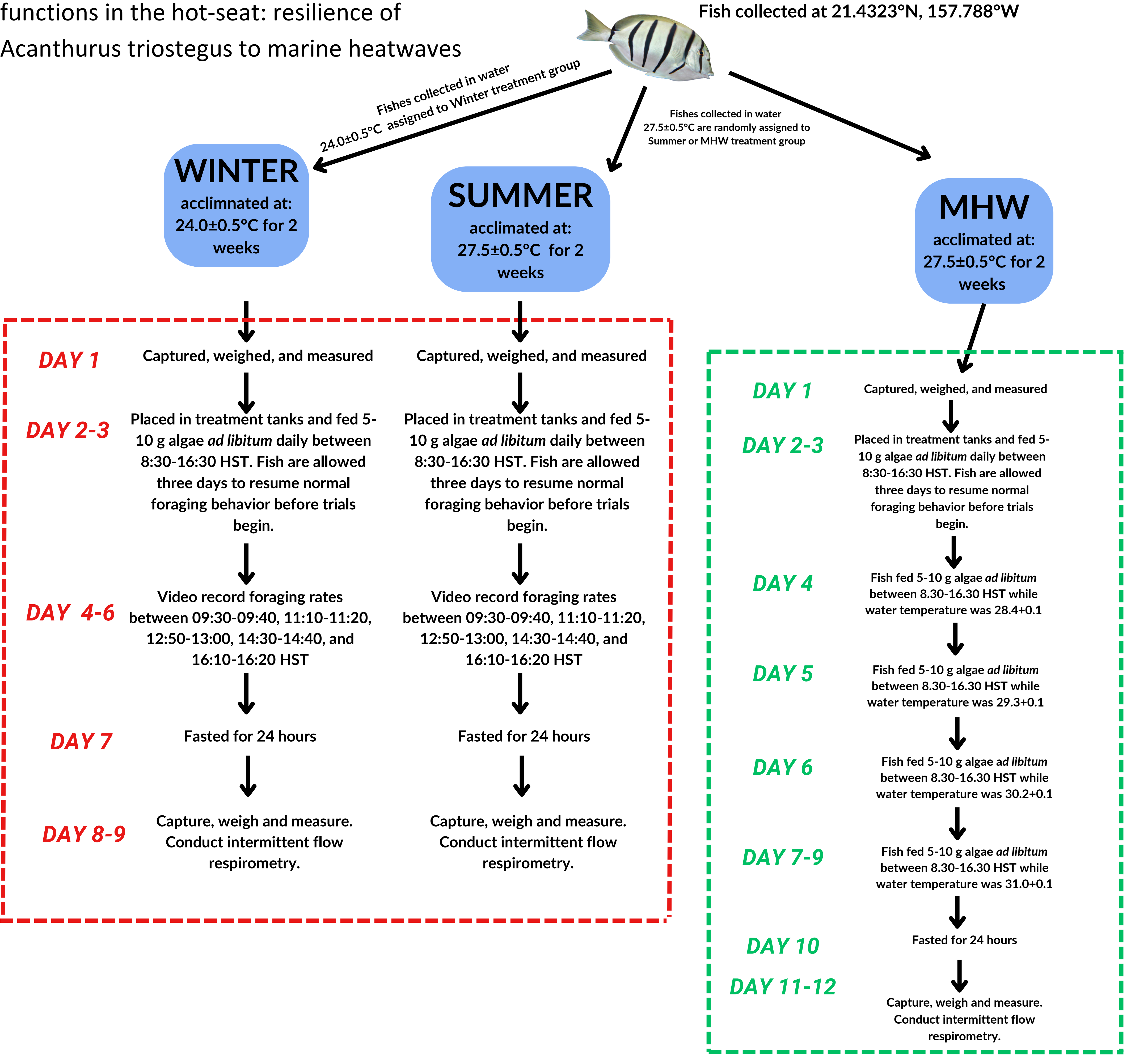

Supplement: S2 Fig — Experimental Set-Up. Detailed diagram of holding tanks for the study on Acanthurus triostegus. The setup includes 4K surveillance cameras operating at 15 frames per second, N842 Series from Lorex Technology Inc., Canada, and two 4–6" PVC structures provided for shelter. An aquarium heater, TH-08005 from Finnex Inc., USA, and a cooling tank with stainless steel coils connected to a water chiller, ECO-1 ½ HP from Ecoplus, USA, are used for temperature regulation. Temperature control is maintained using WH1436 relays from Willhi, China. S4.2. Respirometer Set-Up. Detailed respirometer set-up for A. triostegus to evaluate Standard Metabolic Rate. S4.3. Experimental Timeline. An expanded timeline for the experimental treatments evaluating the resilience of Acanthurus triostegus to marine heatwaves. The timeline includes details of acclimation periods, ramping of tank temperatures for the MHW treatment group, and daily protocols for feeding, video recording, and conducting intermittent-flow respirometry. Fish were subjected to incremental temperature increases up to 31.1 ±0.1°C and provided ~5–10 g of algae daily to assess the impact of thermal stress on their metabolic demands and foraging behavior. (PDF) [file pone.0318410.s002.pdf]
