# Supplementary material for: Herbivore functions in the hot-seat: Resilience of Acanthurus triostegus to marine heatwaves
Source: PLoS One. 2025 Jan 31;20(1):e0318410. doi: 10.1371/journal.pone.0318410 (PMC11785343; doi:10.1371/journal.pone.0318410)
Supplement: S1 File. R-script — (PDF) [file pone.0318410.s004.pdf]

Herbivore functions in the hot-seat: Resilience of *Acanthurus triostegus* to marine heatwaves

Table of Contents

I. Load R packages 2
II. Functions 2
III. Descriptive statistics 3
IV. Body size comparisons 4
V. Model assumptions 5
VI. Data transformation 10
VII. Model comparison 14
VIII. Respecify models 17
IX. Model output 18
X. Model interpretation 19
XI. Model fit 20
XII. Model diagnostics 23
XIII. Planned multiple comparisons 27
XIV. Q_{10} temperature coefficients 28

Preface

Contained in this document is a regression analysis of the physiological and behavioral responses of *Acanthurus triostegus* to marine heatwaves. We fit a group of linear models to our data on the change in body mass, foraging rates (hereafter referred to as mean bite rate), standard metabolic rate (SMR), and activity of *A. triostegus* in response to marine heatwaves. Two modeling approaches were performed and compared here: 1) individual linear regressions for each response variable as a function of marine heatwave treatment and final body mass and 2) a linear mixed model including change in body mass, mean bite rate, and SMR as functions of marine heatwave treatment and final body mass and generalized linear model with a gamma distribution and identity link function for activity as a function marine heatwave and final body mass. These two model structures were then compared using corrected Akaike Information Criterion (AICc) to select the modeling approach for further analysis. Student's t-tests were conducted with a False Discovery Rate correction to compare mean values between temperature treatments. Lastly, Q10 temperature coefficients of SMR were calculated.

I. Load R packages

List of packages used for fitting linear mixed effects models (LMM) and generalized linear models (GLM), adherence to model assumptions, assessing model performance, hypothesis testing, data visualization, & script formatting.

```
require(car)
require(dplyr)
require(effectsize)
require(emmeans)
require(formatR)
require(ggpubr)
require(lmerTest)
require(lme4)
require(MASS)
require(MuMIn)
require(MVN)
require(performance)
require(predictmeans)
require(rmarkdown)
require(rstatix)
require(sjPlot)
require(tidyr)
require(tinytex)
```

II. Functions

Functions written for calculating standard error of the mean (SEM), Q_{10} temperature coefficients, and applying the Box-Cox transformation and Z-score standardization.

```
## Standard error of the mean ----
# x = vector of numerical values
sem <- function(x) {
    stan.error <- sd(x, na.rm = TRUE)/sqrt(length(x[!is.na(x)]))
    return(stan.error)
}

## Q10 temperature coefficient ----
# ri = physiological rate i; ti = temperature i
q10 <- function(r1, r2, t1, t2) {
    q10 = (r2/r1)^(10/(t2 - t1))
    return(q10)
}

## Box-Cox class of power transformations ----
# data = data frame including dependent & independent variables; model =
fitted model object
powerTrans <- function(data, model) {
    if (sum(na.omit(data) > 0) == length(na.omit(data))) {
        bc <- boxCox(model, family = "bcPower", plotit = FALSE)
        l <- bc[[1]][which.max(bc[[2]])]
        print(l)
        print("y > 0, so Box-Cox transformation was used.")
        bcPower(data, l)
```

```
    } else {
        yj <- boxCox(model, family = "yjPower", plotit = FALSE)
        l <- yj[[1]][which.max(yj[[2]])]
        print(l)
        print("y <= 0, so Yeo-Johnson transformation was used.")
        yjPower(data, l)
    }
}

## Z-score standardization ----
# x = vector of numerical values
zstd <- function(x) {
    Z = (x - mean(x, na.rm = TRUE))/sd(x, na.rm = TRUE)
    return(Z)
}
```

III. Descriptive statistics

Histograms of each dependent variable along with descriptive statistics, including variance, standard error of the mean (see section II. for function), minimum & maximum values, and means.

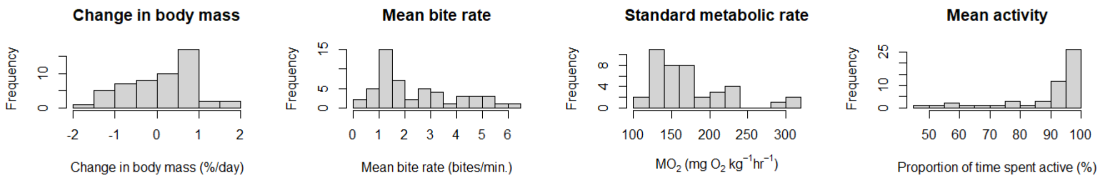


```
## Descriptive statistics ----
# Variance
vars <- aggregate(all_data[, 5:8], by = list(all_data$treatment),
    function(x) var(x, na.rm = TRUE)) %>%
    print()

##    Group.1 change_in_body_mass mean_bite_rate mean_activity       SMR
## 1 Heatwave           0.1671543       2.253060      46.80646 3076.9184
## 2   Summer           0.3035452       1.945643     191.02926  806.4676
## 3   Winter           0.2221165       3.103148     265.49728  185.6296

# Minimum
mins <- aggregate(all_data[, 5:8], by = list(all_data$treatment),
    function(x) min(x, na.rm = TRUE)) %>%
    print()

##    Group.1 change_in_body_mass mean_bite_rate mean_activity      SMR
## 1 Heatwave          -1.6402796    0.600000000      75.20000 161.5656
## 2   Summer          -0.8072507    0.506666667      57.92593 126.2162
## 3   Winter          -0.3006286    0.006666667      47.20000 108.2584

# Maximum
maxs <- aggregate(all_data[, 5:8], by = list(all_data$treatment),
    function(x) max(x, na.rm = TRUE)) %>%
    print()
```

```
##    Group.1 change_in_body_mass mean_bite_rate mean_activity      SMR
## 1 Heatwave          -0.1752541       5.033333     100.00000 311.8198
## 2   Summer           0.8932906       4.626667      97.86667 225.2980
## 3   Winter           1.5479411       6.286667     100.00000 162.5084

# Standard Error of the Mean
sems <- aggregate(all_data[, 5:8], by = list(all_data$treatment),
    function(x) sem(x)) %>%
    print()

##    Group.1 change_in_body_mass mean_bite_rate mean_activity       SMR
## 1 Heatwave          0.09915951      0.3640507      1.659313 16.724827
## 2   Summer          0.15280587      0.3868654      3.833348  8.197904
## 3   Winter          0.10047988      0.3755692      3.473911  3.211348

# Mean
means <- aggregate(all_data[, 5:8], by = list(all_data$treatment),
    function(x) mean(x, na.rm = TRUE)) %>%
    print()

##    Group.1 change_in_body_mass mean_bite_rate mean_activity      SMR
## 1 Heatwave          -0.7952096       2.361961      96.23072 224.4364
## 2   Summer           0.3376984       2.442393      83.99145 173.5190
## 3   Winter           0.6019270       2.443569      88.30505 135.3911
```

IV. Body size comparisons

Body mass was compared between groups before further analysis and revealed that body mass ranges between groups were similar.

```
## Accounting for body mass ----
# Mean body mass of each group
aggregate(all_data$post_trial_body_mass, by = list(all_data$treatment),
    function(x) mean(x))

##    Group.1        x
## 1 Heatwave 46.84471
## 2   Summer 52.00846
## 3   Winter 55.62318

# Standard error of the mean for body mass of each group
aggregate(all_data$post_trial_body_mass, by = list(all_data$treatment),
    function(x) sem(x))

##    Group.1        x
## 1 Heatwave 4.517156
## 2   Summer 5.365146
## 3   Winter 4.093965

# Pairwise comparisons
lm(data = all_data, post_trial_body_mass ~ treatment) %>%
    emmeans(., pairwise ~ treatment, adjust = "fdr", nesting = NULL)

## $emmeans
##  treatment emmean   SE df lower.CL upper.CL
##  Heatwave    46.8 4.62 49     37.6     56.1
```

```
##  Summer       52.0 5.28 49     41.4     62.6
##  Winter       55.6 4.06 49     47.5     63.8
##
## Confidence level used: 0.95
##
## $contrasts
##  contrast          estimate   SE df t.ratio p.value
##  Heatwave - Summer    -5.16 7.02 49  -0.736  0.5900
##  Heatwave - Winter    -8.78 6.15 49  -1.427  0.4798
##  Summer - Winter      -3.61 6.66 49  -0.542  0.5900
##
## P value adjustment: fdr method for 3 tests
```

V. Model assumptions

Assessing dependent variables for univariate & multivariate normality & homoscedasticity to ensure adherence to parametric model assumptions. Covariance between dependent variables were calculated to create a covariance matrix. Normality was assessed using Shapiro-Wilk's test and by visually examining quantile-quantile plots. After assessing the assumption of normality, homoscedasticity was assessed using parametric (i.e. Bartlett's test) and non-parametric tests (i.e. Levene's test), and spread plots of model residuals against fitted values were also visually examined to determine homoscedasticity among groups.

```
## Covariance between dependent variables
cor(all_data[, 5:8], use = "complete.obs") %>%
    round(digits = 3)

##                      change_in_body_mass mean_bite_rate mean_activity    SMR
## change_in_body_mass                1.000          0.467         0.063 -0.641
## mean_bite_rate                     0.467          1.000         0.420 -0.156
## mean_activity                      0.063          0.420         1.000  0.134
## SMR                               -0.641         -0.156         0.134  1.000

## Fit linear models to test univariate normality & homoscedasticity
models <- paste(names(all_data)[6:8], "model", sep = "_")
# Fit mean bite rate, mean activity, and SMR models
for (i in 1:length(models)) {
    assign(models[i], lm(all_data[, i + 5] ~ treatment +
post_trial_body_mass,
        all_data))
}
# Fit change in body mass model
assign("change_in_body_mass_model", lm(change_in_body_mass ~
    treatment, all_data))

## Mean bite rate ----
# Shapiro-Wilk's test for Normality
shapiro.test(residuals(mean_bite_rate_model))

##
##  Shapiro-Wilk normality test
##
## data:  residuals(mean_bite_rate_model)
## W = 0.92116, p-value = 0.002069
```

```r
# Plots & Bartlett's test for homoscedasticity
plot(mean_bite_rate_model, main = "Mean bite rate")
```

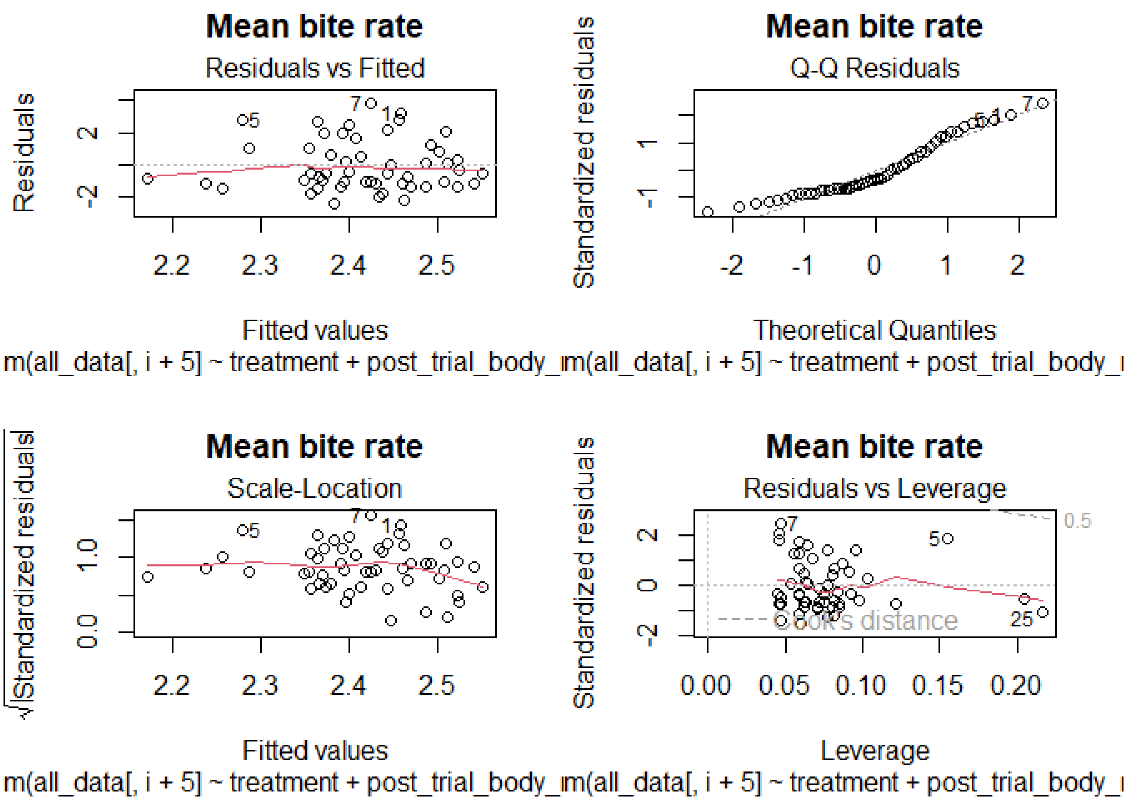


```r
bartlett.test(mean_bite_rate ~ treatment, data = all_data)

##
##  Bartlett test of homogeneity of variances
##
## data:  mean_bite_rate by treatment
## Bartlett's K-squared = 0.92531, df = 2, p-value = 0.6296

## Standard metabolic rate ---- Shapiro-Wilk's test for
## Normality
shapiro.test(residuals(SMR_model))

##
##  Shapiro-Wilk normality test
##
## data:  residuals(SMR_model)
## W = 0.97079, p-value = 0.3658

# Plots & Bartlett's test for homoscedasticity
plot(SMR_model, main = "Standard metabolic rate")
```

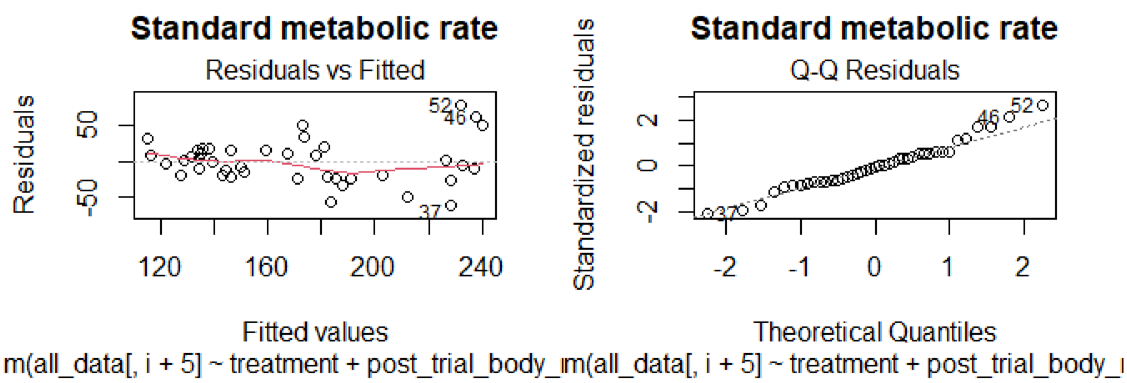

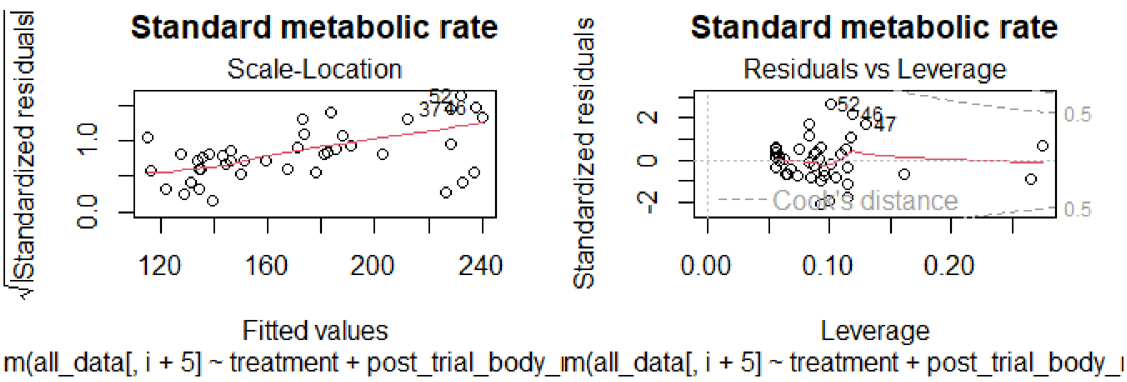


```
bartlett.test(SMR ~ treatment, data = all_data)

##
##  Bartlett test of homogeneity of variances
##
## data:  SMR by treatment
## Bartlett's K-squared = 23.4, df = 2, p-value = 8.294e-06

## Mean activity ---- Shapiro-Wilk's test for Normality
shapiro.test(residuals(mean_activity_model))

##
##  Shapiro-Wilk normality test
##
## data:  residuals(mean_activity_model)
## W = 0.80444, p-value = 7.578e-07

# Plots & Levene's test for homoscedasticity
plot(mean_activity_model, main = "Mean activity")
```

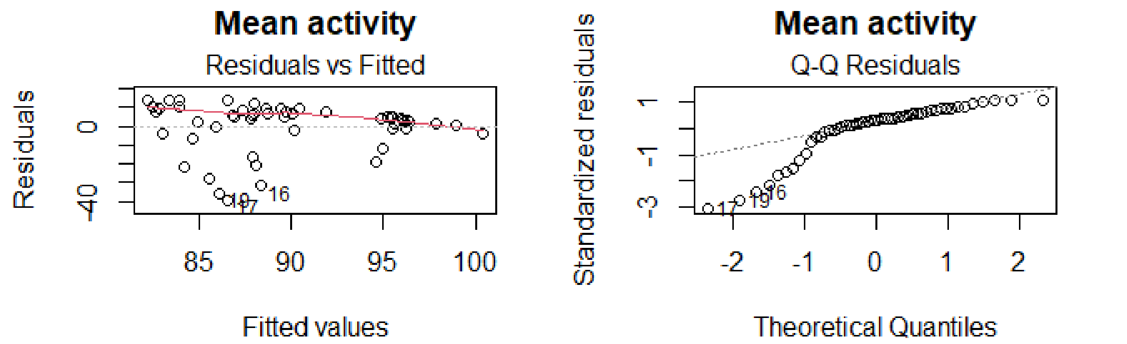

m(all_data[, i + 5] ~ treatment + post_trial_body_ m(all_data[, i + 5] ~ treatment + post_trial_body_

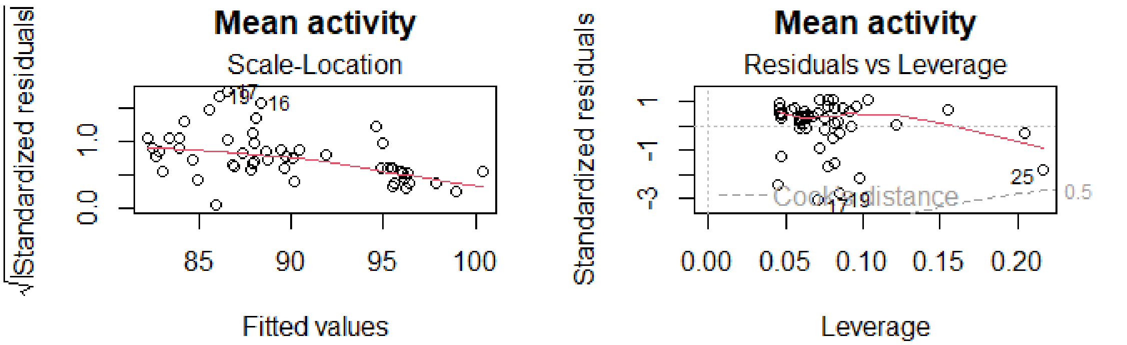

m(all_data[, i + 5] ~ treatment + post_trial_body_ m(all_data[, i + 5] ~ treatment + post_trial_body_

```
leveneTest(mean_activity ~ treatment, data = all_data)

## Levene's Test for Homogeneity of Variance (center = median)
##       Df F value Pr(>F)
## group  2  1.9102 0.1589
##       49

## Change in body mass data ---- Shapiro-Wilk's test for
## Normality
shapiro.test(residuals(change_in_body_mass_model))

##
##  Shapiro-Wilk normality test
##
## data:  residuals(change_in_body_mass_model)
## W = 0.98372, p-value = 0.693

# Plots & Bartlett's test of homoscedasticity
plot(change_in_body_mass_model, main = "Change in body mass")
```

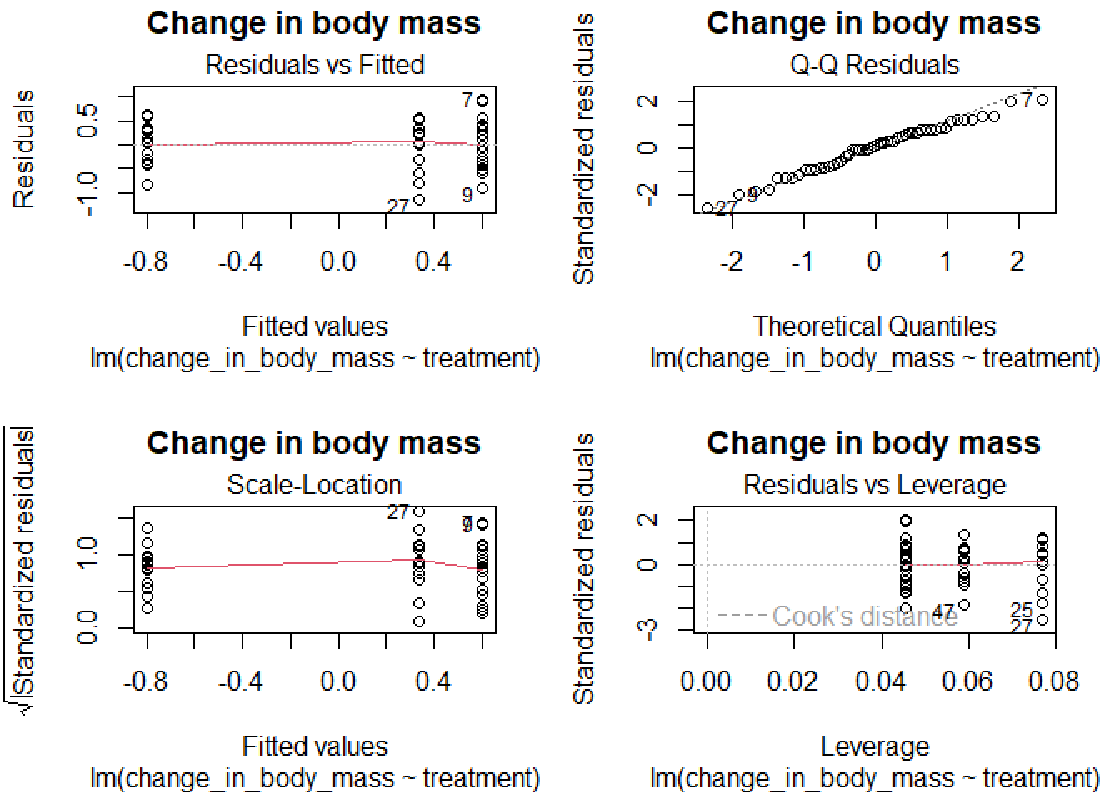


```r
bartlett.test(change_in_body_mass ~ treatment, data = all_data)

##
##  Bartlett test of homogeneity of variances
##
## data:  change_in_body_mass by treatment
## Bartlett's K-squared = 1.204, df = 2, p-value = 0.5477

# Multivariate normality tests & quantile-quantile plots
mvn(LMM_data[, c(5, 6, 8)], mvnTest = "mardia", univariateTest = "SW")

## $multivariateNormality
##              Test        Statistic             p value Result
## 1 Mardia Skewness  27.3816096688188 0.00226561158272275     NO
## 2 Mardia Kurtosis 0.801432182860381   0.422881490075538    YES
## 3             MVN              <NA>                <NA>     NO
##
## $univariateNormality
##           Test            Variable Statistic   p value Normality
## 1 Shapiro-Wilk change_in_body_mass    0.9734    0.4415    YES
## 2 Shapiro-Wilk    mean_bite_rate      0.9009    0.0018    NO
## 3 Shapiro-Wilk         SMR            0.8566    0.0001    NO
##
## $Descriptives
##                      n        Mean   Std.Dev      Median         Min        Max
## change_in_body_mass 41   0.1983868  0.731996   0.3345377  -1.6402796   1.547941
## mean_bite_rate      41   2.4018609  1.539779   1.8333333   0.3133333   6.286667
## SMR                 41 170.4406956 49.267936 155.7716401 108.2583670 311.819825
```

```
##                             25th        75th       Skew   Kurtosis
## change_in_body_mass  -0.4406055   0.6977047 -0.3583174 -0.5541718
## mean_bite_rate        1.3333333   3.2955556  0.8477243 -0.3801665
## SMR                 136.7614719 187.8413138  1.3206693  1.1722858
```

VI. Data transformation

Mean bite rate and activity data were transformed using a square root transformation and log10 transformation for negative skew to adhere to the assumption of normality. Standard metabolic rate data were transformed using the Box-Cox transformation to adhere to the assumption of homoscedasticity (see Section II for Box-Cox transformation function). Linear models were fit to transformed data to reassess the assumptions of normality and homoscedasticity. The model for change in body mass was not refit as the data adhered to both assumptions of normality and homoscedasticity.

Individual linear models were fit to each response to estimate the relationship between the heatwave treatments and final body mass for mean bite rate, standard metabolic rate, and mean activity. For mean activity. The parametric approach had a better model fit and was the final model used in our analysis. A linear model was fit to the change in body mass data to estimate the relationship with our heatwave treatments, but final body mass was omitted as a predictor because the change in body mass is calculated using final body mass.

```
## Mean bite rate - square root transformation
all_data$mean_bite_rate_sqrt <- sqrt(all_data$mean_bite_rate)

## Standard metabolic rate - Box-Cox transformation
all_data$SMR_BC <- powerTrans(all_data$SMR, SMR_model)

## [1] -1.2
## [1] "y > 0, so Box-Cox transformation was used."

## Mean activity - log10 for negative skew
all_data$mean_activity_trans <- with(all_data, log10(max(mean_activity +
    1) - mean_activity))  # *Highest values become the lowest values and vice
versa*

## Reassess model assumptions with transformed data Refit
## mean bite rate model
mean_bite_rate_sqrt_model <- lm(mean_bite_rate_sqrt ~ treatment +
    post_trial_body_mass, all_data)
# Shapiro-Wilk's test for Normality
shapiro.test(residuals(mean_bite_rate_sqrt_model))

##
##  Shapiro-Wilk normality test
##
## data:  residuals(mean_bite_rate_sqrt_model)
## W = 0.97732, p-value = 0.4193

# Plots & Bartlett's test for homoscedasticity
plot(mean_bite_rate_sqrt_model, mean = "SQRT(Mean Bite Rate)")
```

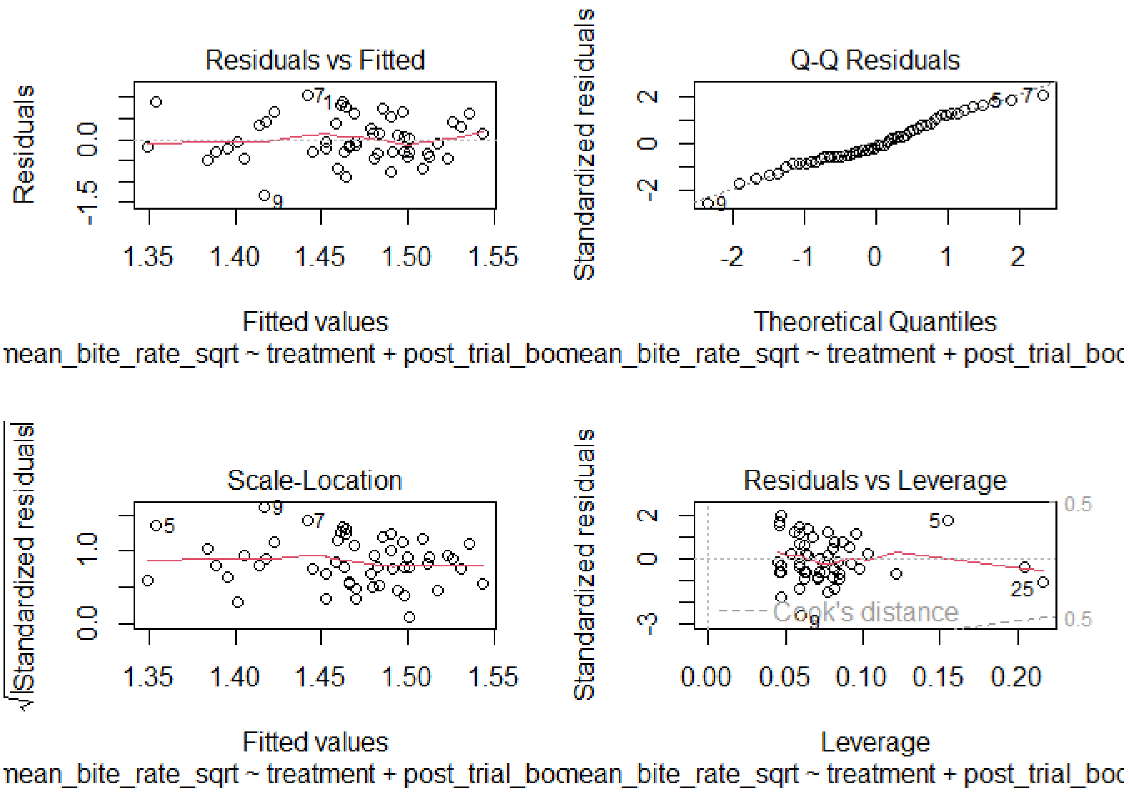


```r
bartlett.test(mean_bite_rate_sqrt ~ treatment, data = all_data)

##
##  Bartlett test of homogeneity of variances
##
## data:  mean_bite_rate_sqrt by treatment
## Bartlett's K-squared = 1.0425, df = 2, p-value = 0.5938

# ANOVA table
Anova(mean_bite_rate_sqrt_model, test = "F", type = "II")

## Anova Table (Type II tests)
##
## Response: mean_bite_rate_sqrt
##                       Sum Sq Df F value Pr(>F)
## treatment             0.0155  2  0.0273 0.9731
## post_trial_body_mass  0.0889  1  0.3118 0.5792
## Residuals            13.6819 48

# Refit SMR model
SMR_BC_model <- lm(SMR_BC ~ treatment + post_trial_body_mass,
    all_data)
# Shapiro-Wilk's test for Normality
shapiro.test(residuals(SMR_BC_model))

##
##  Shapiro-Wilk normality test
##
## data:  residuals(SMR_BC_model)
## W = 0.97452, p-value = 0.478
```

```
# Plots & Bartlett's test for homoscedasticity
plot(SMR_BC_model, main = "Box-Cox(Standard metabolic rate)")
```

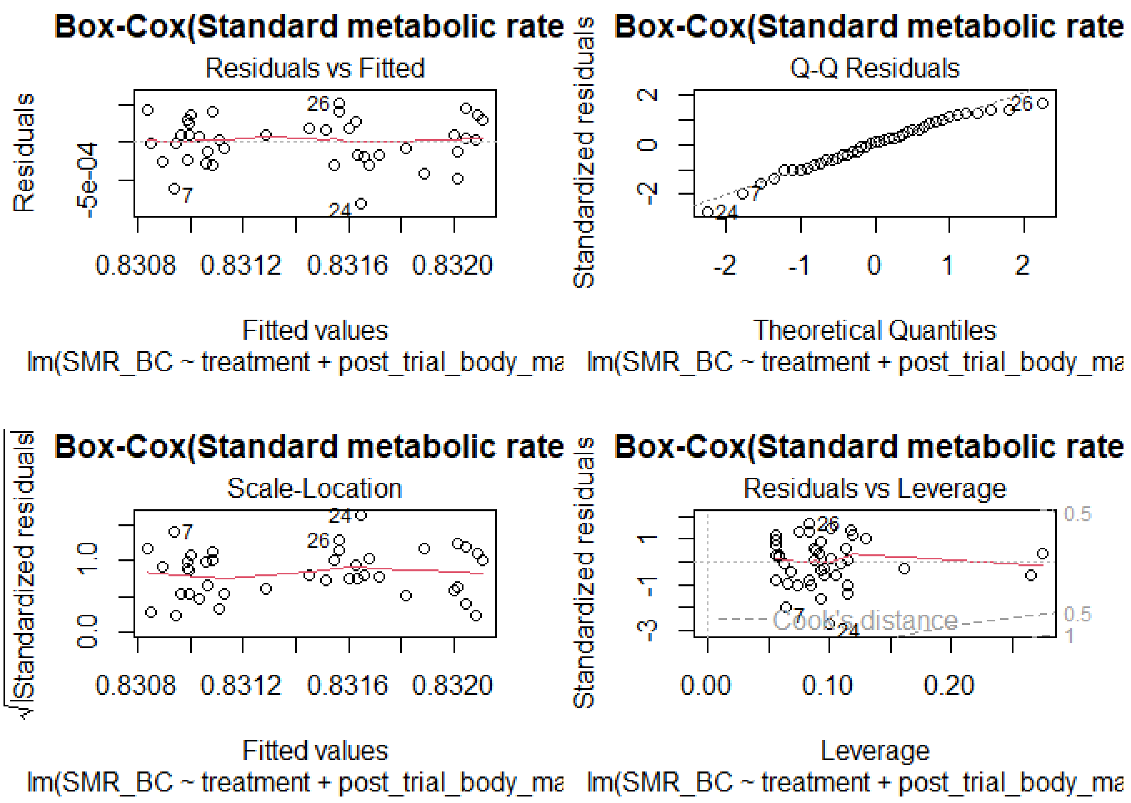


```
bartlett.test(SMR_BC ~ treatment, data = all_data)

##
##  Bartlett test of homogeneity of variances
##
## data:  SMR_BC by treatment
## Bartlett's K-squared = 1.0052, df = 2, p-value = 0.605

# ANOVA table
Anova(SMR_BC_model, test = "F", type = "II")

## Anova Table (Type II tests)
##
## Response: SMR_BC
##                         Sum Sq Df F value    Pr(>F)
## treatment           6.9582e-06  2 34.1550 3.945e-09 ***
## post_trial_body_mass 4.0950e-07  1  4.0204   0.05231 .
## Residuals           3.7689e-06 37
## ---
## Signif. codes:  0 '***' 0.001 '**' 0.01 '*' 0.05 '.' 0.1 ' ' 1

# Refit activity model
mean_activity_trans_model <- lm(mean_activity_trans ~ treatment +
    post_trial_body_mass, all_data)
# Shapiro-Wilk's test for Normality
shapiro.test(residuals(mean_activity_trans_model))

##
##  Shapiro-Wilk normality test
```

```
##
## data:  residuals(mean_activity_trans_model)
## W = 0.97109, p-value = 0.2349

# Plots & Bartlett's test for homoscedasticity
plot(mean_activity_trans_model, main = "Mean activity")
```

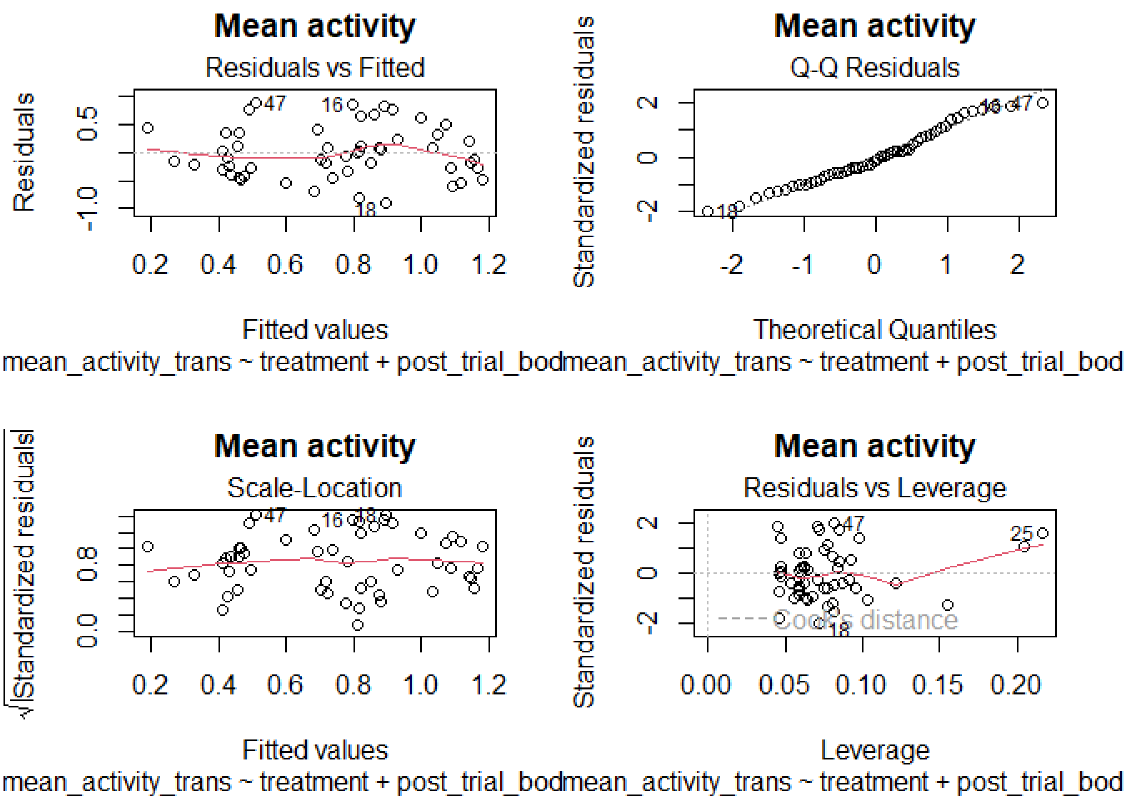


```
bartlett.test(mean_activity_trans ~ treatment, data = all_data)

##
##  Bartlett test of homogeneity of variances
##
## data:  mean_activity_trans by treatment
## Bartlett's K-squared = 1.8878, df = 2, p-value = 0.3891

# ANOVA table
Anova(mean_activity_trans_model, test = "F", type = "III")

## Anova Table (Type III tests)
##
## Response: mean_activity_trans
##                      Sum Sq Df F value    Pr(>F)
## (Intercept)          2.1847  1  9.9309 0.0027986 **
## treatment            3.6031  2  8.1893 0.0008709 ***
## post_trial_body_mass 0.3616  1  1.6437 0.2059753
## Residuals           10.5595 48
## ---
## Signif. codes:  0 '***' 0.001 '**' 0.01 '*' 0.05 '.' 0.1 ' ' 1
```

For our second modeling approach, a Z-score standardization was applied to the data for change in body mass, mean bite rate, & SMR (see section II. for function). This technique standardizes the values in terms of standard deviation units & can take on positive, negative, & 0 values, indicating an individual value's standard deviation from the group mean. Z-score standardization also standardizes the scale of these different data sets needed for multivariate linear modeling. A Yeo-Johnson transformation was applied to the mean bite rate & SMR data following the Z-score standardization to adhere to the assumption of normality (see section II. for function). The Yeo-Johnson transformation is derived from the Box-Cox transformation but can be applied to negative values.

```
# Yeo-Johnson Transformations
LMM_zdata$mean_bite_rate_BC <- powerTrans(LMM_zdata$mean_bite_rate,
    mean_bite_rate_model)

## [1] -1.2
## [1] "y <= 0, so Yeo-Johnson transformation was used."

LMM_zdata$SMR_BC <- powerTrans(LMM_zdata$SMR, SMR_model)

## [1] -1.2
## [1] "y <= 0, so Yeo-Johnson transformation was used."
```

VII. Model comparison

Two different modeling approaches were considered in this study and compared using corrected Akaike Information Criterion (AICc). The first modeling approach used a simple linear regression for each response variable (i.e. change in body mass, mean bite rate, standard metabolic rate, and mean activity) where marine heatwave treatment and final body mass were used as predictor variables except for the change in body mass model. The second modelling approach used a linear mixed effects model for change in body mass, mean bite rate, and standard metabolic rate including a random intercept for each individual fish to account for the non-independence of these data since they were all measured in the same individuals. Overall, the simple linear regressions had the lowest AICc values and were selected for further analysis.

```
## Change in body mass, mean bite rate, & SMR model ----
## Fit the linear mixed effects model
lmodel <- lmer(values ~ response + response:treatment +
response:post_trial_body_mass +
    (1 | id), data = LMM_zdata)
summary(lmodel)

## Linear mixed model fit by REML. t-tests use Satterthwaite's method [
## lmerModLmerTest]
## Formula:
## values ~ response + response:treatment + response:post_trial_body_mass +
##     (1 | id)
##    Data: LMM_zdata
##
## REML criterion at convergence: 438.2
##
## Scaled residuals:
##     Min      1Q  Median      3Q     Max
## -4.1071 -0.3499  0.0533  0.5192  1.6550
```

```
##
## Random effects:
##  Groups   Name        Variance Std.Dev.
##  id       (Intercept) 0.2137   0.4622
##  Residual             0.8750   0.9354
## Number of obs: 145, groups:  id, 52
##
## Fixed effects:
##                                                Estimate Std. Error
## (Intercept)                                   -0.823402   0.445398
## responsemean_bite_rate_BC                      0.504381   0.564709
## responseSMR_BC                                 1.436782   0.640283
## responsechange_in_body_mass:treatmentSummer    1.489548   0.386548
## responsemean_bite_rate_BC:treatmentSummer      0.171639   0.386548
## responseSMR_BC:treatmentSummer                -0.492377   0.432804
## responsechange_in_body_mass:treatmentWinter    1.852423   0.343867
## responsemean_bite_rate_BC:treatmentWinter      0.004253   0.343867
## responseSMR_BC:treatmentWinter                -1.906086   0.396008
## responsechange_in_body_mass:post_trial_body_mass -0.006417   0.007824
## responsemean_bite_rate_BC:post_trial_body_mass   -0.010468   0.007824
## responseSMR_BC:post_trial_body_mass           -0.006200   0.008914
##                                                       df t value Pr(>|t|)
## (Intercept)                                   122.746360  -1.849 0.066909
## responsemean_bite_rate_BC                      80.110608   0.893 0.374441
## responseSMR_BC                                 89.726708   2.244 0.027294
## responsechange_in_body_mass:treatmentSummer   122.746359   3.853 0.000187
## responsemean_bite_rate_BC:treatmentSummer     122.746359   0.444 0.657803
## responseSMR_BC:treatmentSummer                129.105178  -1.138 0.257376
## responsechange_in_body_mass:treatmentWinter   122.746359   5.387 3.51e-07
## responsemean_bite_rate_BC:treatmentWinter     122.746359   0.012 0.990152
## responseSMR_BC:treatmentWinter                130.119137  -4.813 4.05e-06
## responsechange_in_body_mass:post_trial_body_mass 122.746360  -0.820 0.413746
## responsemean_bite_rate_BC:post_trial_body_mass   122.746360  -1.338 0.183395
## responseSMR_BC:post_trial_body_mass           129.756110  -0.696 0.487966
##
## (Intercept)                                   .
## responsemean_bite_rate_BC
## responseSMR_BC                                *
## responsechange_in_body_mass:treatmentSummer   ***
## responsemean_bite_rate_BC:treatmentSummer
## responseSMR_BC:treatmentSummer
## responsechange_in_body_mass:treatmentWinter   ***
## responsemean_bite_rate_BC:treatmentWinter
## responseSMR_BC:treatmentWinter                ***
## responsechange_in_body_mass:post_trial_body_mass
## responsemean_bite_rate_BC:post_trial_body_mass
## responseSMR_BC:post_trial_body_mass
## ---
## Signif. codes:  0 '***' 0.001 '**' 0.01 '*' 0.05 '.' 0.1 ' ' 1
##
## Correlation of Fixed Effects:
##             (Intr) rs___BC rsSMR_BC r___:S r___BC:S rSMR_BC:S r___:W r___BC:W
## rspnsm___BC -0.634
## rspnsSMR_BC -0.559  0.441
## rspnsc___:S -0.286  0.181   0.160
## rspn___BC:S -0.056 -0.181   0.000    0.196
## rspSMR_BC:S -0.050  0.000  -0.288    0.175  0.175
## rspnsc___:W -0.254  0.161   0.142    0.503  0.099    0.088
```

```
## rspn___BC:W -0.050 -0.161   0.000    0.099  0.503    0.088     0.196
## rspSMR_BC:W -0.043  0.000  -0.337    0.086  0.086    0.566     0.170  0.170
## rspn___:___ -0.823  0.522   0.460   -0.105 -0.021   -0.018    -0.200 -0.039
## rs___BC:___ -0.161 -0.522   0.000   -0.021 -0.105   -0.018    -0.039 -0.200
## rSMR_BC:___ -0.142  0.000  -0.587   -0.018 -0.018   -0.038    -0.034 -0.034
##             rSMR_BC:W r___:_ r___BC:_
## rspnsm___BC
## rspnsSMR_BC
## rspnsc___:S
## rspn___BC:S
## rspSMR_BC:S
## rspnsc___:W
## rspn___BC:W
## rspSMR_BC:W
## rspn___:___ -0.034
## rs___BC:___ -0.034     0.196
## rSMR_BC:___ -0.021     0.172  0.172
```

```
# Compare linear mixed effects model to individual models
# using AICc
AICc(lmodel, change_in_body_mass_model, mean_bite_rate_sqrt_model,
    SMR_BC_model)
```

```
##                            df       AICc
## lmodel                     14  469.45624
## change_in_body_mass_model   4   75.55883
## mean_bite_rate_sqrt_model   5   89.44496
## SMR_BC_model                5 -536.22687
```

```
# Fit a generalized linear model to mean activity data
activity_glm <- glm(mean_activity ~ treatment * post_trial_body_mass,
    data = all_data, family = Gamma(link = "identity"))
summary(activity_glm)
```

```
##
## Call:
## glm(formula = mean_activity ~ treatment * post_trial_body_mass,
##     family = Gamma(link = "identity"), data = all_data)
##
## Coefficients:
##                                      Estimate Std. Error t value Pr(>|t|)
## (Intercept)                           90.0108     9.3793   9.597 1.48e-12 ***
## treatmentSummer                       16.6979    13.1438   1.270   0.2103
## treatmentWinter                      -23.6444    12.4225  -1.903   0.0633 .
## post_trial_body_mass                   0.1328     0.1907   0.696   0.4897
## treatmentSummer:post_trial_body_mass  -0.5702     0.2441  -2.336   0.0239 *
## treatmentWinter:post_trial_body_mass   0.2619     0.2405   1.089   0.2817
## ---
## Signif. codes:  0 '***' 0.001 '**' 0.01 '*' 0.05 '.' 0.1 ' ' 1
##
## (Dispersion parameter for Gamma family taken to be 0.02024113)
##
##     Null deviance: 1.5338  on 51  degrees of freedom
## Residual deviance: 1.0898  on 46  degrees of freedom
## AIC: 426.99
##
## Number of Fisher Scoring iterations: 5
```

```r
# Compare models using AICc
AICc(mean_activity_trans_model, activity_glm)

##                           df      AICc
## mean_activity_trans_model  5  75.97454
## activity_glm               7 429.53930
```

VIII. Respecify models

The individual linear regressions for mean bite rate and SMR were refit to remove the interaction term between treatment and final body mass.

```r
# Refit mean bite rate model
mean_bite_rate_sqrt_model <- lm(mean_bite_rate_sqrt ~ treatment +
    post_trial_body_mass, all_data)
summary(mean_bite_rate_sqrt_model)

## 
## Call:
## lm(formula = mean_bite_rate_sqrt ~ treatment + post_trial_body_mass, 
##     data = all_data)
## 
## Residuals:
##      Min       1Q   Median       3Q      Max 
## -1.33472 -0.31781 -0.08369  0.38530  1.06579 
## 
## Coefficients:
##                       Estimate Std. Error t value Pr(>|t|)    
## (Intercept)           1.567749   0.227901   6.879 1.13e-08 ***
## treatmentSummer       0.044128   0.197789   0.223    0.824    
## treatmentWinter       0.009252   0.175950   0.053    0.958    
## post_trial_body_mass -0.002235   0.004003  -0.558    0.579    
## ---
## Signif. codes:  0 '***' 0.001 '**' 0.01 '*' 0.05 '.' 0.1 ' ' 1
## 
## Residual standard error: 0.5339 on 48 degrees of freedom
## Multiple R-squared:  0.007566,   Adjusted R-squared:  -0.05446 
## F-statistic: 0.122 on 3 and 48 DF,  p-value: 0.9467

# Refit SMR model
SMR_BC_model <- lm(SMR_BC ~ treatment + post_trial_body_mass,
    all_data)
summary(SMR_BC_model)

## 
## Call:
## lm(formula = SMR_BC ~ treatment + post_trial_body_mass, data = all_data)
## 
## Residuals:
##        Min         1Q     Median         3Q        Max 
## -8.220e-04 -1.877e-04  3.111e-05  2.371e-04  5.197e-04 
## 
## Coefficients:
##                        Estimate Std. Error  t value Pr(>|t|)    
## (Intercept)           8.323e-01  1.663e-04 5003.423  < 2e-16 ***
```

```
## treatmentSummer      -4.110e-04  1.333e-04   -3.083  0.00386 **
## treatmentWinter      -9.846e-04  1.222e-04   -8.060 1.15e-09 ***
## post_trial_body_mass -5.511e-06  2.748e-06   -2.005  0.05231 .
## ---
## Signif. codes:  0 '***' 0.001 '**' 0.01 '*' 0.05 '.' 0.1 ' ' 1
##
## Residual standard error: 0.0003192 on 37 degrees of freedom
##   (11 observations deleted due to missingness)
## Multiple R-squared:  0.662,  Adjusted R-squared:  0.6346
## F-statistic: 24.15 on 3 and 37 DF,  p-value: 7.869e-09
```

```
# Refit activity model
mean_activity_trans_model <- lm(mean_activity_trans ~ treatment +
    post_trial_body_mass, all_data)
summary(mean_activity_trans_model)
```

```
##
## Call:
## lm(formula = mean_activity_trans ~ treatment + post_trial_body_mass,
##     data = all_data)
##
## Residuals:
##      Min       1Q   Median       3Q      Max
## -0.89493 -0.31252 -0.06515  0.32489  0.90044
##
## Coefficients:
##                       Estimate Std. Error t value Pr(>|t|)
## (Intercept)           0.630944   0.200215   3.151 0.002799 **
## treatmentSummer       0.688888   0.173760   3.965 0.000244 ***
## treatmentWinter       0.416663   0.154575   2.696 0.009660 **
## post_trial_body_mass -0.004509   0.003517  -1.282 0.205975
## ---
## Signif. codes:  0 '***' 0.001 '**' 0.01 '*' 0.05 '.' 0.1 ' ' 1
##
## Residual standard error: 0.469 on 48 degrees of freedom
## Multiple R-squared:  0.2608, Adjusted R-squared:  0.2146
## F-statistic: 5.644 on 3 and 48 DF,  p-value: 0.002142
```

IX. Model output

Model outputs were assessed visually by comparing observations to predicted values from the models for each response variable. Scatter plots are individual dependent variables plotted against treatment. Blue & black circles represent observed & predicted values, respectively. Solid circles are mean values with standard error bars, and semitransparent circles are the individual data points.

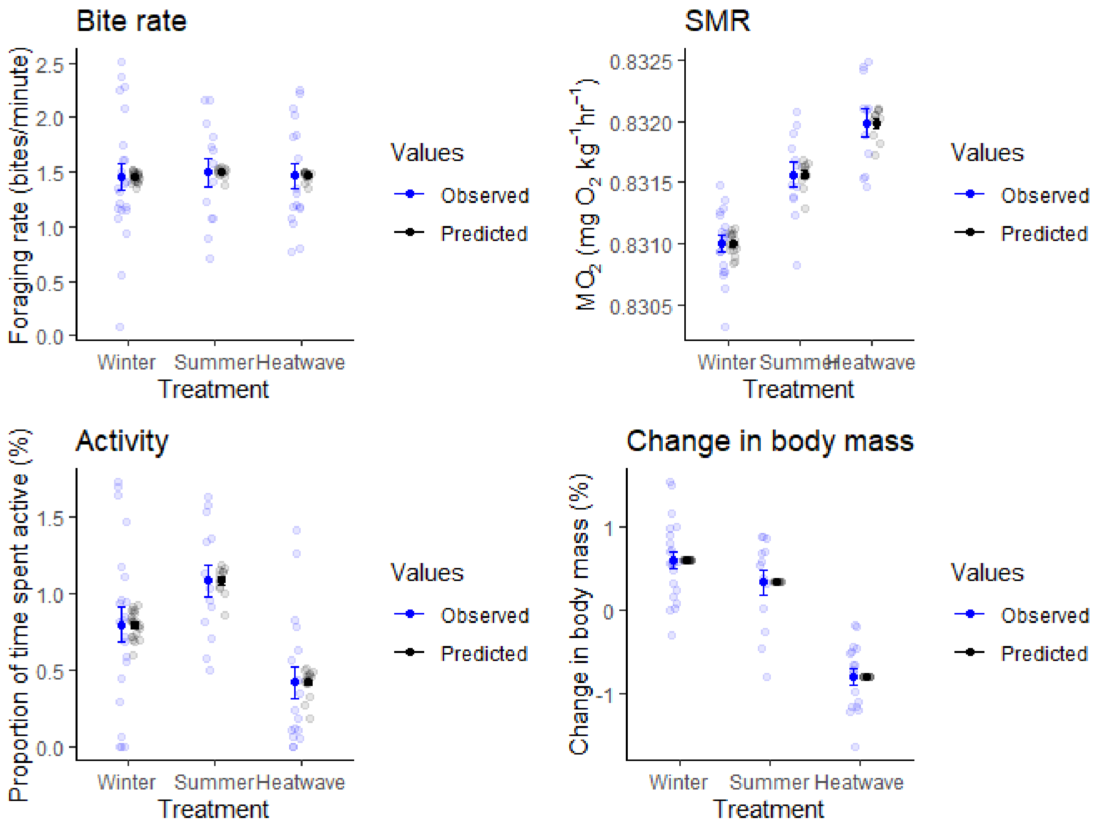

X. Model interpretation

Estimated marginal means & slopes against mass were extracted from the models using the "emmeans" package. Standard errors, degrees of freedom, & 95% confidence intervals for each coefficient are also included.

```
## Mean bite rate ----
# Extract estimated slope values for mass
emtrends(mean_bite_rate_sqrt_model, ~treatment, var = "post_trial_body_mass")

##  treatment post_trial_body_mass.trend    SE df lower.CL  upper.CL
##  Heatwave                     -0.00224 0.004 48  -0.0103  0.00581
##  Summer                       -0.00224 0.004 48  -0.0103  0.00581
##  Winter                       -0.00224 0.004 48  -0.0103  0.00581
##
## Confidence level used: 0.95

# Extract estimated marginal means
emmeans(mean_bite_rate_sqrt_model, specs = "treatment")

##  treatment emmean    SE df lower.CL upper.CL
##  Heatwave    1.45 0.131 48     1.19     1.72
##  Summer      1.50 0.148 48     1.20     1.79
##  Winter      1.46 0.115 48     1.23     1.69
##
## Confidence level used: 0.95

## Standard metabolic rate ----
# Extract estimated slope values for mass
emtrends(SMR_BC_model, ~treatment, var = "post_trial_body_mass")
```

```
##  treatment post_trial_body_mass.trend       SE df  lower.CL upper.CL
##  Heatwave                    -5.51e-06 2.75e-06 37 -1.11e-05  5.8e-08
##  Summer                      -5.51e-06 2.75e-06 37 -1.11e-05  5.8e-08
##  Winter                      -5.51e-06 2.75e-06 37 -1.11e-05  5.8e-08
##
## Confidence level used: 0.95

# Extract estimated marginal means
emmeans(SMR_BC_model, specs = "treatment")

##  treatment  emmean         SE df lower.CL upper.CL
##  Heatwave  0.83198 9.6251e-05 37  0.83179  0.83218
##  Summer    0.83157 9.2172e-05 37  0.83138  0.83176
##  Winter    0.83100 7.5229e-05 37  0.83084  0.83115
##
## Confidence level used: 0.95

## Mean activity ----
# Extract estimated slope values for mass
emtrends(mean_activity_trans_model, ~treatment, var = "post_trial_body_mass")

##  treatment post_trial_body_mass.trend      SE df lower.CL upper.CL
##  Heatwave                     -0.00451 0.00352 48  -0.0116  0.00256
##  Summer                       -0.00451 0.00352 48  -0.0116  0.00256
##  Winter                       -0.00451 0.00352 48  -0.0116  0.00256
##
## Confidence level used: 0.95

# Extract estimated marginal means
emmeans(mean_activity_trans_model, specs = "treatment")

##  treatment emmean    SE df lower.CL upper.CL
##  Heatwave   0.397 0.115 48    0.166    0.629
##  Summer     1.086 0.130 48    0.824    1.348
##  Winter     0.814 0.101 48    0.611    1.017
##
## Confidence level used: 0.95

## Change in body mass ----
# Extract estimated marginal means
emmeans(mean_activity_trans_model, specs = "treatment")

##  treatment emmean    SE df lower.CL upper.CL
##  Heatwave   0.397 0.115 48    0.166    0.629
##  Summer     1.086 0.130 48    0.824    1.348
##  Winter     0.814 0.101 48    0.611    1.017
##
## Confidence level used: 0.95
```

XI. Model fit

Model fit was assessed using adjusted R^2 values from the model output. Effect sizes were considered by comparing model coefficients between treatments.

```r
## R-squared ----

# Mean bite rate
summary(mean_bite_rate_sqrt_model)

##
## Call:
## lm(formula = mean_bite_rate_sqrt ~ treatment + post_trial_body_mass,
##     data = all_data)
##
## Residuals:
##      Min       1Q   Median       3Q      Max
## -1.33472 -0.31781 -0.08369  0.38530  1.06579
##
## Coefficients:
##                       Estimate Std. Error t value Pr(>|t|)
## (Intercept)           1.567749   0.227901   6.879 1.13e-08 ***
## treatmentSummer       0.044128   0.197789   0.223    0.824
## treatmentWinter       0.009252   0.175950   0.053    0.958
## post_trial_body_mass -0.002235   0.004003  -0.558    0.579
## ---
## Signif. codes:  0 '***' 0.001 '**' 0.01 '*' 0.05 '.' 0.1 ' ' 1
##
## Residual standard error: 0.5339 on 48 degrees of freedom
## Multiple R-squared:  0.007566,   Adjusted R-squared:  -0.05446
## F-statistic: 0.122 on 3 and 48 DF,  p-value: 0.9467

# Standard metabolic rate
summary(SMR_BC_model)

##
## Call:
## lm(formula = SMR_BC ~ treatment + post_trial_body_mass, data = all_data)
##
## Residuals:
##        Min         1Q     Median         3Q        Max
## -8.220e-04 -1.877e-04  3.111e-05  2.371e-04  5.197e-04
##
## Coefficients:
##                        Estimate Std. Error  t value Pr(>|t|)
## (Intercept)           8.323e-01  1.663e-04 5003.423  < 2e-16 ***
## treatmentSummer      -4.110e-04  1.333e-04   -3.083  0.00386 **
## treatmentWinter      -9.846e-04  1.222e-04   -8.060 1.15e-09 ***
## post_trial_body_mass -5.511e-06  2.748e-06   -2.005  0.05231 .
## ---
## Signif. codes:  0 '***' 0.001 '**' 0.01 '*' 0.05 '.' 0.1 ' ' 1
##
## Residual standard error: 0.0003192 on 37 degrees of freedom
##   (11 observations deleted due to missingness)
## Multiple R-squared:  0.662,  Adjusted R-squared:  0.6346
## F-statistic: 24.15 on 3 and 37 DF,  p-value: 7.869e-09
```

```r
# Change in body mass
summary(change_in_body_mass_model)

##
## Call:
## lm(formula = change_in_body_mass ~ treatment, data = all_data)
##
## Residuals:
##      Min       1Q   Median       3Q      Max
## -1.14495 -0.36481  0.04282  0.34781  0.94601
##
## Coefficients:
##                 Estimate Std. Error t value Pr(>|t|)
## (Intercept)      -0.7952     0.1148  -6.926 8.64e-09 ***
## treatmentSummer   1.1329     0.1744   6.495 4.02e-08 ***
## treatmentWinter   1.3971     0.1529   9.139 3.66e-12 ***
## ---
## Signif. codes:  0 '***' 0.001 '**' 0.01 '*' 0.05 '.' 0.1 ' ' 1
##
## Residual standard error: 0.4734 on 49 degrees of freedom
## Multiple R-squared:  0.6441, Adjusted R-squared:  0.6296
## F-statistic: 44.35 on 2 and 49 DF,  p-value: 1.014e-11

# Mean activity
summary(mean_activity_trans_model)

##
## Call:
## lm(formula = mean_activity_trans ~ treatment + post_trial_body_mass,
##     data = all_data)
##
## Residuals:
##      Min       1Q   Median       3Q      Max
## -0.89493 -0.31252 -0.06515  0.32489  0.90044
##
## Coefficients:
##                       Estimate Std. Error t value Pr(>|t|)
## (Intercept)           0.630944   0.200215   3.151 0.002799 **
## treatmentSummer       0.688888   0.173760   3.965 0.000244 ***
## treatmentWinter       0.416663   0.154575   2.696 0.009660 **
## post_trial_body_mass -0.004509   0.003517  -1.282 0.205975
## ---
## Signif. codes:  0 '***' 0.001 '**' 0.01 '*' 0.05 '.' 0.1 ' ' 1
##
## Residual standard error: 0.469 on 48 degrees of freedom
## Multiple R-squared:  0.2608, Adjusted R-squared:  0.2146
## F-statistic: 5.644 on 3 and 48 DF,  p-value: 0.002142
```

XII. Model diagnostics

The models were diagnosed for multicollinearity, outlier effects, & the presence of influential observations. Multicollinearity in models with multiple predictors (i.e. mean bite rate, standard metabolic rate, and mean activity) was assessed by calculating variance inflation factors (VIF). The presence of influential observations was assessed visually using leverage plots & plots of Cook's distance for each observation. An ANOVA was performed for each model to test the effect of the model predictors on the dependent variables.

```
## Mean bite rate ----
# Variance inflation factor
vif(mean_bite_rate_sqrt_model)

##                         GVIF Df GVIF^(1/(2*Df))
## treatment           1.041579  2        1.010237
## post_trial_body_mass 1.041579  1        1.020578

# Diagnostic plots
plot(mean_bite_rate_sqrt_model)
# ANOVA table
Anova(mean_bite_rate_sqrt_model, test = "F", type = "II")

## Anova Table (Type II tests)
##
## Response: mean_bite_rate_sqrt
##                      Sum Sq Df F value Pr(>F)
## treatment            0.0155  2  0.0273 0.9731
## post_trial_body_mass 0.0889  1  0.3118 0.5792
## Residuals           13.6819 48

## Standard metabolic rate ----
# Variance inflation factor
vif(SMR_BC_model)

##                         GVIF Df GVIF^(1/(2*Df))
## treatment           1.001311  2        1.000328
## post_trial_body_mass 1.001311  1        1.000655

# Diagnostic plots
plot(SMR_BC_model)
# ANOVA table
Anova(SMR_BC_model, test = "F", type = "II")

## Anova Table (Type II tests)
##
## Response: SMR_BC
##                         Sum Sq Df F value    Pr(>F)
## treatment           6.9582e-06  2 34.1550 3.945e-09 ***
## post_trial_body_mass 4.0950e-07  1  4.0204   0.05231 .
## Residuals           3.7689e-06 37
## ---
## Signif. codes:  0 '***' 0.001 '**' 0.01 '*' 0.05 '.' 0.1 ' ' 1
```

```r
## Mean activity ----
# Variance inflation factor
vif(mean_activity_trans_model)

##                             GVIF Df GVIF^(1/(2*Df))
## treatment               1.041579  2        1.010237
## post_trial_body_mass 1.041579  1        1.020578

# Diagnostic plots
plot(mean_activity_trans_model)
# ANOVA table
Anova(mean_activity_trans_model, test = "F", type = "II")

## Anova Table (Type II tests)
##
## Response: mean_activity_trans
##                          Sum Sq Df F value    Pr(>F)
## treatment                3.6031  2  8.1893 0.0008709 ***
## post_trial_body_mass     0.3616  1  1.6437 0.2059753
## Residuals               10.5595 48
## ---
## Signif. codes:  0 '***' 0.001 '**' 0.01 '*' 0.05 '.' 0.1 ' ' 1

## Change in body mass ----
# Variance inflation factor could not be calculated for change in body mass
because the model contains only 1 predictor term

# Diagnostic plots
plot(change_in_body_mass_model)
# ANOVA table
Anova(change_in_body_mass_model, test = "F", type = "II")

## Anova Table (Type II tests)
##
## Response: change_in_body_mass
##            Sum Sq Df F value    Pr(>F)
## treatment 19.878  2  44.349 1.014e-11 ***
## Residuals 10.982 49
## ---
## Signif. codes:  0 '***' 0.001 '**' 0.01 '*' 0.05 '.' 0.1 ' ' 1
```

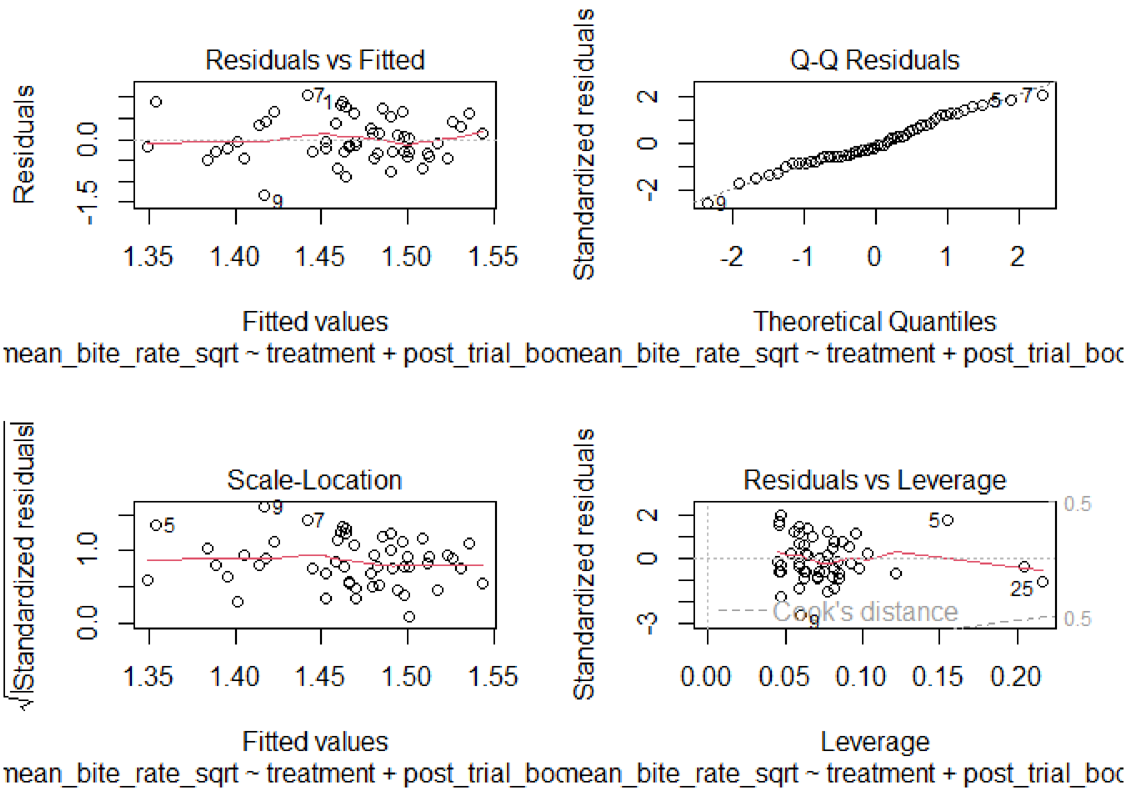
Residuals vs Fitted
Residuals
Fitted values
mean_bite_rate_sqrt ~ treatment + post_trial_bo
Q-Q Residuals
Standardized residuals
Theoretical Quantiles
mean_bite_rate_sqrt ~ treatment + post_trial_bo
Scale-Location
√|Standardized residuals|
Fitted values
mean_bite_rate_sqrt ~ treatment + post_trial_bo
Residuals vs Leverage
Standardized residuals
Cook's distance
Leverage
mean_bite_rate_sqrt ~ treatment + post_trial_bo

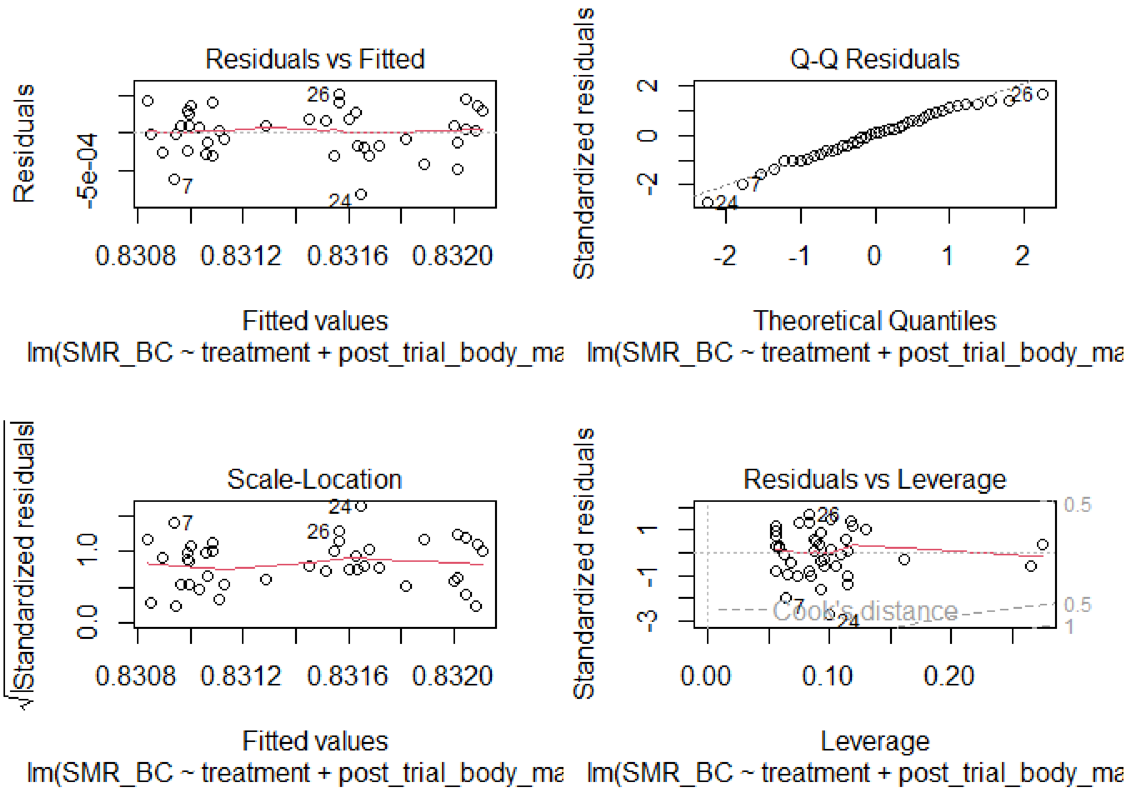
Residuals vs Fitted
Residuals
Fitted values
lm(SMR_BC ~ treatment + post_trial_body_ma
Q-Q Residuals
Standardized residuals
Theoretical Quantiles
lm(SMR_BC ~ treatment + post_trial_body_ma
Scale-Location
√|Standardized residuals|
Fitted values
lm(SMR_BC ~ treatment + post_trial_body_ma
Residuals vs Leverage
Standardized residuals
Cook's distance
Leverage
lm(SMR_BC ~ treatment + post_trial_body_ma

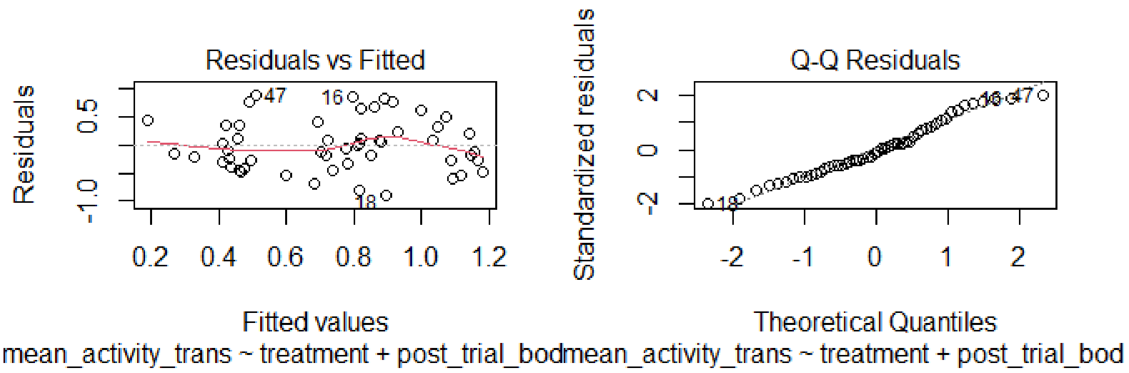

Residuals vs Fitted
Residuals
Fitted values
mean_activity_trans ~ treatment + post_trial_bod
Q-Q Residuals
Standardized residuals
Theoretical Quantiles
mean_activity_trans ~ treatment + post_trial_bod
Scale-Location
√|Standardized residuals|
Fitted values
mean_activity_trans ~ treatment + post_trial_bod
Residuals vs Leverage
Standardized residuals
Cook's distance
Leverage
mean_activity_trans ~ treatment + post_trial_bod

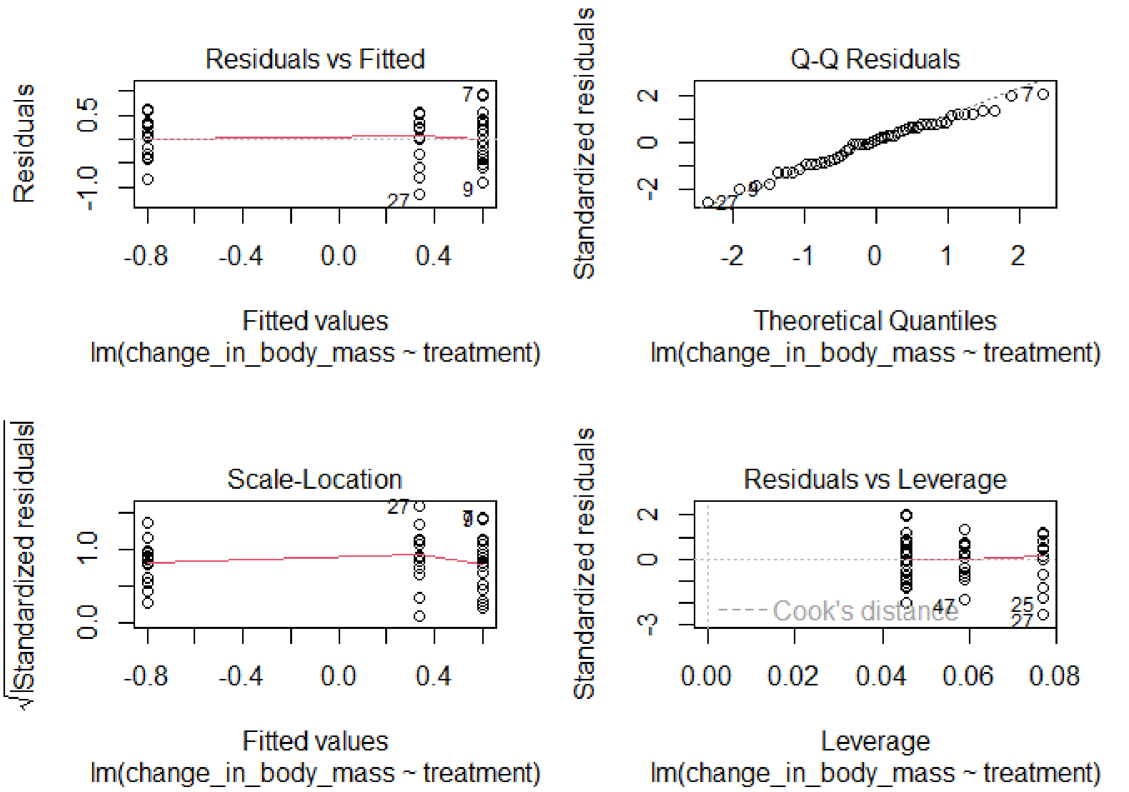

Residuals vs Fitted
Residuals
Fitted values
lm(change_in_body_mass ~ treatment)
Q-Q Residuals
Standardized residuals
Theoretical Quantiles
lm(change_in_body_mass ~ treatment)
Scale-Location
√|Standardized residuals|
Fitted values
lm(change_in_body_mass ~ treatment)
Residuals vs Leverage
Standardized residuals
Cook's distance
Leverage
lm(change_in_body_mass ~ treatment)

XIII. Planned multiple comparisons

Estimated marginal means from the two models were extracted & compared among treatments using a two-sample *t*-test. False discovery rate (fdr) adjustments were made to control for the family-wise error rate.

```
## Compute contrasts with FDR adjustment ----
# Mean bite rate
emmeans(mean_bite_rate_sqrt_model, pairwise ~ treatment, adjust = "fdr")

## $emmeans
##  treatment emmean    SE df lower.CL upper.CL
##  Heatwave    1.45 0.131 48     1.19     1.72
##  Summer      1.50 0.148 48     1.20     1.79
##  Winter      1.46 0.115 48     1.23     1.69
##
## Confidence level used: 0.95
##
## $contrasts
##  contrast          estimate    SE df t.ratio p.value
##  Heatwave - Summer -0.04413 0.198 48  -0.223  0.9583
##  Heatwave - Winter -0.00925 0.176 48  -0.053  0.9583
##  Summer - Winter    0.03488 0.187 48   0.186  0.9583
##
## P value adjustment: fdr method for 3 tests

# Standard metabolic rate
emmeans(SMR_BC_model, pairwise ~ treatment, adjust = "fdr")

## $emmeans
##  treatment  emmean         SE df lower.CL upper.CL
##  Heatwave  0.83198 9.6251e-05 37  0.83179  0.83218
##  Summer    0.83157 9.2172e-05 37  0.83138  0.83176
##  Winter    0.83100 7.5229e-05 37  0.83084  0.83115
##
## Confidence level used: 0.95
##
## $contrasts
##  contrast          estimate       SE df t.ratio p.value
##  Heatwave - Summer 0.000411 0.000133 37   3.083  0.0039
##  Heatwave - Winter 0.000985 0.000122 37   8.060  <.0001
##  Summer - Winter   0.000574 0.000119 37   4.820  <.0001
##
## P value adjustment: fdr method for 3 tests

# Mean activity
emmeans(mean_activity_trans_model, pairwise ~ treatment, adjust = "fdr")

## $emmeans
##  treatment emmean    SE df lower.CL upper.CL
##  Heatwave   0.397 0.115 48    0.166    0.629
##  Summer     1.086 0.130 48    0.824    1.348
##  Winter     0.814 0.101 48    0.611    1.017
##
## Confidence level used: 0.95
##
```

```
## $contrasts
##  contrast            estimate    SE df t.ratio p.value
##  Heatwave - Summer    -0.689 0.174 48  -3.965  0.0007
##  Heatwave - Winter    -0.417 0.155 48  -2.696  0.0145
##  Summer - Winter       0.272 0.165 48   1.654  0.1046
##
## P value adjustment: fdr method for 3 tests
```

```
# Change in body mass
emmeans(change_in_body_mass_model, pairwise ~ treatment, adjust = "fdr")
```

```
## $emmeans
##  treatment emmean    SE df lower.CL upper.CL
##  Heatwave  -0.795 0.115 49  -1.0259   -0.564
##  Summer     0.338 0.131 49   0.0738    0.602
##  Winter     0.602 0.101 49   0.3991    0.805
##
## Confidence level used: 0.95
##
## $contrasts
##  contrast            estimate    SE df t.ratio p.value
##  Heatwave - Summer    -1.133 0.174 49  -6.495  <.0001
##  Heatwave - Winter    -1.397 0.153 49  -9.139  <.0001
##  Summer - Winter      -0.264 0.166 49  -1.595  0.1170
##
## P value adjustment: fdr method for 3 tests
```

XIV. Q_{10} temperature coefficients

```
##     t1   t2 group1   group2      q10
## 1 24.0 27.5 Winter   Summer 2.031783
## 2 27.5 31.0 Summer Heatwave 2.085809
## 3 24.0 31.0 Winter Heatwave 2.058619
```
